# Direct determination of phenolic secoiridoids in olive oil by ultra-high performance liquid chromatography-triple quadruple mass spectrometry analysis

**DOI:** 10.1038/s41598-019-52060-5

**Published:** 2019-10-29

**Authors:** Antonio Luque-Muñoz, Ruben Tapia, Ali Haidour, Jose Justicia, Juan M. Cuerva

**Affiliations:** 1 Nuclear Magnetic Resonance Unit, Scientific Instrumentation Center, University of Granada, E-18071 Granada, Spain; 2 Department of Organic Chemistry, University of Granada, Campus Fuentenueva s/n, E-18071 Granada, Spain

**Keywords:** Analytical chemistry, Chemical synthesis

## Abstract

In recent years, a large number of biological properties and an important role in the organoleptic characteristics of olive oil have been attributed to phenolic secoiridoids, such as oleacein, oleocanthal, oleuropein aglycone and ligstroside aglycone. Consequently, quantifying them is of great interest for the olive oil sector. Currently, there is no consensus in which analytical method must be use to accurately determine these compounds in olive oil, mainly owing to the lack of reference standards for calibration. In this work, analytical standards of phenolic secoiridoids have been used to develop a quantitative and rapid analytical method by UHPLC-MS/MS, in which sample extraction is not carried out. Simple dilutions of the sample with dry tetrahydrofuran and dry acetonitrile were performed before analysing them. It is worth noting that under these conditions the generation of artefacts such as acetals and hemiacetals of the aldehydic forms is highly reduced. The detection and quantification was performed with a Xevo TQS tandem quadrupole mass spectrometer. The method was validated at four concentration levels and finally applied to six samples of extra virgin olive oil.

## Introduction

Olive oil is a basic component of the Mediterranean diet whose production has been developed in the Mediterranean countries and in recent decades it has developed in other parts of the world^1^. Olive oil contains two fractions, saponifiable (approximately 98% of the total composition) and unsaponifiable. The main compounds of the saponifiable fraction are triglycerides of fatty acids with oleic acid in the highest proportion (55–83%). In the unsaponifiable fraction there are more than 200 different compounds such as sterols, triterpenic alcohols, tocopherols and phenols^2^. Oleic acid and phenolic compounds are responsible for health-protecting activities and nutritional and nutraceutical properties of the olive oil. Due to this, on November 2004, the Federal Drug Administration of the USA released a claim concerning the benefits of daily ingestion of olive oil thanks to the high amount of monounsaturated fatty oleic acid^3^ and, on May 2012, European Union authorized the claim “olive oil polyphenols contribute to the protection of blood lipids from oxidative stress” for olive oil which “contains at least 5 mg of hydroxytyrosol and its derivatives (eg. oleuropein complex and tyrosol) per 20 g of olive oil”^4^.

**Figure 1:**
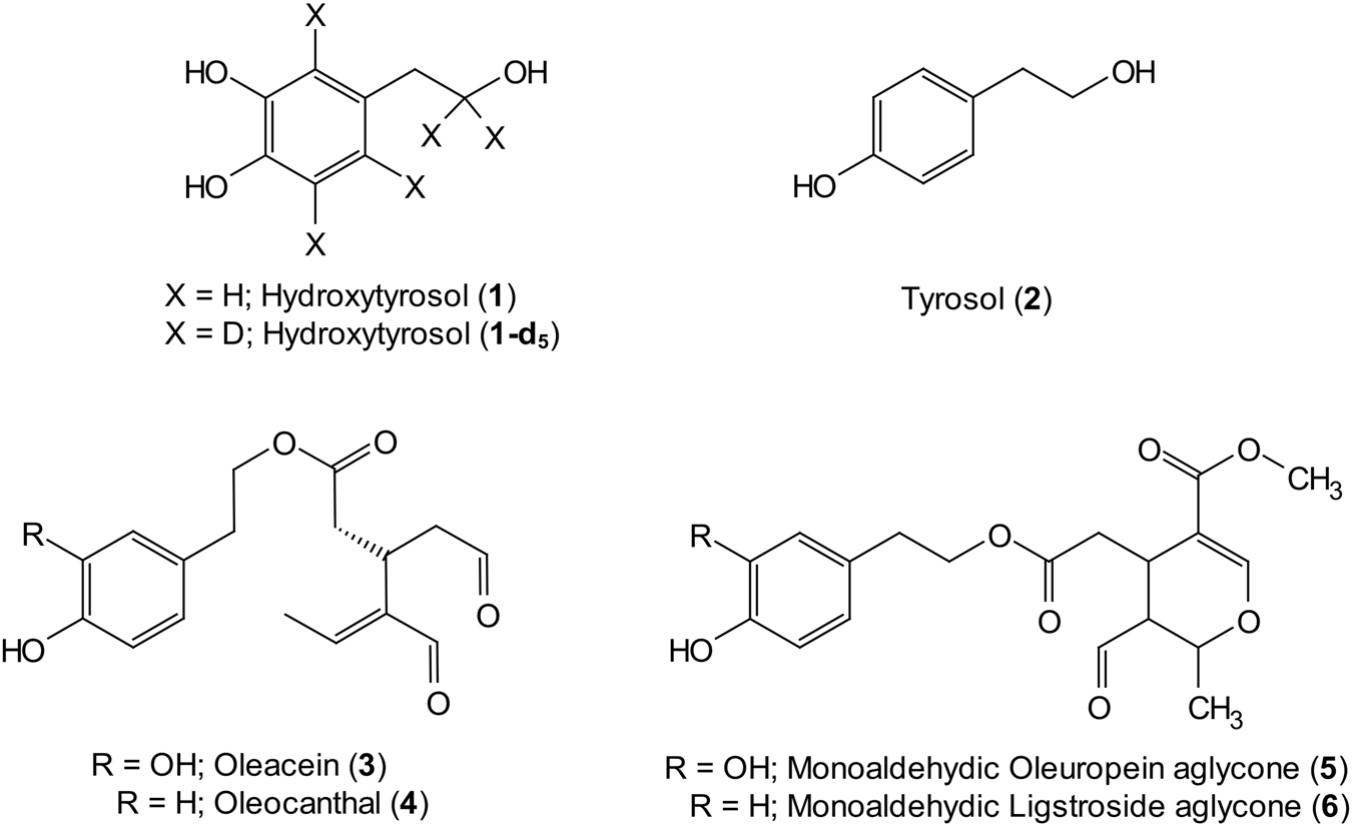
Structure of compounds **1** to **6**.

In addition to the hydroxytyrosol (3,4-DHPEA, **1**) derivatives, at least the tyrosol (*p*-HPEA, **2**) derivatives should also be considered in the calculation of “olive oil polyphenols” content to support the health claim of EU^5^. All these derivatives are classified as secoiridoids (or phenolic secoiridoids) which represent the most abundant subclass of phenolic compounds in olive oil. Monoaldehydic forms of oleuropein (3,4-DHPEA-EA, **5**) and ligstroside aglycone (*p*-HPEA-EA, **6**), and the dialdehydic forms their decarboxymethylated derivatives, oleacein (3,4-DHPEA-EDA, **3**) and oleocanthal (*p*-HPEA-EDA, **4**) (Fig. 1) are the main secoiridoids in olive oil. Besides the monoaldehydic forms, there are more oleuropein and ligstroside aglycone isomers, some of them are also found in olive oil^6–10^.

EEC Regulation 2568/91 establishes analytical and organoleptic parameters to define the quality grade of an olive oil. Conforming to the results of chemical and sensory analyses, an olive oil is classified as extra virgin olive oil (EVOO), virgin olive oil or lampante olive oil. The positive attributes of olive oil tasting, pungency and bitterness, are related to phenolic compounds and especially to secoiridoids^11–14^.

An analytical method to determine oleuropein and ligstroside aglycone isomers, oleacein and oleocanthal can allow to verify the health claim of EU in the labelling of an olive oil and be of assistance, together with the sensory tasting panel, to classify olive oil. A study has shown the “variability of judgment in the organoleptic evaluation” in common commercial EVOOs^15^.

Several analytical methods have been reported in the last two decades to jointly, individually or even with other phenols analyse these four compounds in olive oil using a large number of analytical techniques^10,16–27^. In recent years, reverse phase liquid chromatography with high and low resolution mass detection has been widely used. As far as we know, all the analytical methods developed by liquid chromatography (except one in which direct injection is used and oleacein, oleocanthal, oleuropein aglycone and ligstroside aglycone are quantified in terms of hydroxytyrosol and tyrosol^28^) are based on the extraction of the phenolic fraction of olive oil using ACN, MeOH or mixtures with water. Monohydrates, methyl hemiacetals and dimethyl acetals are formed during the extraction owing to the reactivity of the mentioned secoiridoids with water and MeOH. Although these compounds can be reconverted in their corresponding aldehydic forms by evaporating the extract to dryness, Celano *et al*. proved that the conversion is not always complete^22^. Nuclear magnetic resonance (NMR) in aprotic solvent has been used to determine secoiridoids in olive oil without using extraction methods^20^ but it requires extensive analysis times, unless DPFGSE techniques are not used^10,29–31^. Moreover, to implement NMR as a routine technique in any analytical laboratory is expensive since the maintenance cost of NMR spectrometers is high.

On the other hand, most analytical methods use purified EVOO phenolic extracts^32,33^ because of the lack of reference standards for calibration so currently, there is no standardized analytical method for the accurate determination of secoiridoids in olive oil. In its stead, International Olive Council (IOC) proposed a quantitative analysis by HPLC-UV using syringic acid as internal standard to express the total phenol content of olive oil as tyrosol equivalents^34^. However, this method is inaccurate because each phenolic compound gives a different response under UV detection at 280 nm^22^. An indirect method consisting of acid hydrolysis of the phenolic secoiridoids and subsequent determination of tyrosol and hydroxytyrosol by LC-UV^35,36^ or LC-MS^37^, has also been used for supporting the health claim in virgin olive oils. Nevertheless, the biological properties of secoiridoids^38–45^ are different and cannot be summarized by the amount of hydroxytyrosol and tyrosol equivalents. An especial case is oleocanthal which has anti-inflammatory, antioxidant and antimicrobial activities, anticancer properties and neuroprotective effects^46^. Moreover, the chemical reactivity of each component can be reflected in different conversions altering this kind of quantification. A selective method is therefore mandatory. Recently, our group has tried to avoid these drawbacks developing a selective method for the determination of **3** and **5** using deuterated surrogates^16^.

In this paper is presented a new and simple analytical method to directly measure the concentration of oleuropein and ligstroside aglycone isomers, oleacein and oleocanthal in olive oil by UHPLC-MS/MS. The objectives of the proposed method are: a) to minimize and even eliminate the formation of monohydrates, hemiacetals and acetals obtained during the preparation of the sample, which could potentially interfere in the determination and b) increase the accuracy of the method using analytical standards.

## Methods

### Chemicals and reagents

Oleacein (**3**), oleocanthal (**4**), monoaldehydic forms of oleuropein (**5**) and ligstroside (**6**) aglycone and hydroxytyrosol-d_5_ (**1-d**_**5**_) used as internal standard (IS), were supplied by Vadolivo S.A. (Jaén, Spain). The synthesis and purification of the analytes are available in Supplementary Information. Each monoaldehydic aglycone is a mixture of four diastereoisomers (see Supplementary Table S1-S2): (5S, 8R, 9S), (5S, 8S, 9S), (5S, 8S, 9R) and (5S, 8R, 9R). NMR spectra of the five compounds are shown in Supplementary Figs S1–S12. Individual standard solutions of compounds and working standard mixtures were prepared in dry acetonitrile (ACN) and stored at −20 °C in amber glass vials sealed with septum and under argon stream. Dry acetonitrile and dry tetrahydrofuran (THF) used for the preparation of standards and samples were provided by Panreac (Darmstadt, Germany). Water (18.2 MΩ·cm) used for the preparation mobile phases was purified using a Milli-Q system from Millipore (Bedford, MA, USA). The rest of solvents used for the preparation of mobile phases, LC–MS grade ACN, ammonia (≥25%) and formic acid (≥98%) and HPLC grade THF, were purchased from Sigma-Aldrich (St. Louis, MO, USA).

### Instrumentation and software

UHPLC–MS/MS analyses were performed using a Waters Acquity UPLC^TM^ H–Class system (Waters, Manchester, UK). The mass spectrometer was a Xevo TQS tandem quadrupole mass spectrometer (Waters) equipped with StepWave ion guide and an orthogonal Z–spray electrospray ionization (ESI) source. Pursuit XRs Ultra C18 column (2.8 μm; 2 × 100 mm) (Agilent, CA, USA) was selected for the separation of compounds. Statgraphics Centurion XVI v.16.2 (Statpoint Technologies Inc., VA, USA) and Microsoft Excel 2013 v.15.0 (Microsoft, WA, USA) were used for the statistical treatment of data. MassLynx v.4.1 software was used for UHPLC-MS instrument control, peak detection and integration.

### Collecting and preparing olive oil samples

A total of 6 different EVOOs were collected from different supermarkets located in Southern Spain. Samples were stored in 50 ml amber glass bottles in the dark at 4 °C until analysis.

**Figure 2:**
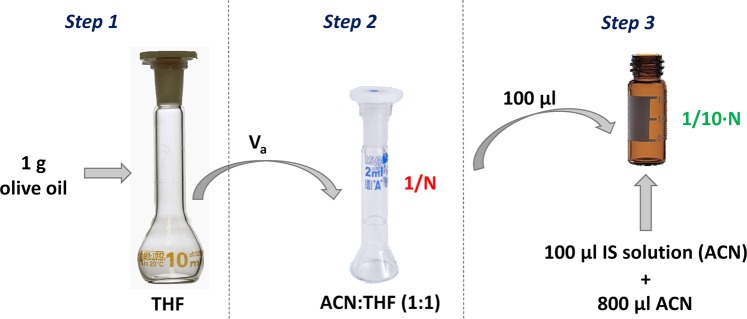
Dilution process of the sample. V_a_ (ml) is aliquot volume and N, dilution factor, is equal to 2/V_a_.

The samples were diluted in three steps before being analysed by UHPLC-MS/MS. In the fisrt dilution, 1.000 g of olive oil was added to a 10 mL volumetric flask which was filled with dry THF. Next, an aliquot (V_a_) was diluted with a dry ACN:dry THF (1:1, v/v) mixture to 2 mL in volumetric flask. Finally, 100 μL of the previous solution was mixed in a chromatography vial with 100 μL of an IS solution in dry ACN (300 μg L^−1^) and 800 μL dry ACN. The dilution process is shown in Fig. 2.

### Ultra-high performance liquid chromatography–tandem mass spectrometry conditions

Chromatographic separation was performed under isocratic conditions, using a binary solvent mixture consisting of water (solvent A, 30%) and ACN (solvent B, 70%) at a flow rate of 300 μL min^−1^. Column cleaning after each run and before equilibration was subsequently performed using 100% ACN or THF, according to the program described in Supplementary Table S3. ACN:THF (1:1, v/v) mixture was used as needle wash solvent. Injection volume was 10 μL and the column was thermostated at 30 °C.

Electrospray ionization (ESI) was used as ion source in the mass spectrometer (MS). Negative ion mode was employed and [M-H]^−^ was selected as precursor ion in all compounds. For increased sensitivity and selectivity, triple quadrupole mass spectrometry (QqQ) was operated in multiple reaction monitoring (MRM) mode. The optimum cone voltage to maximize formation of [M-H]^−^ and the optimal collision energies that give rise to two most sensitive MRM transitions (the first for quantification and the second for confirmation, see Table 1) were obtained for each compound by direct infusion electrospray ionization mass spectrometry of standard solution prepared in dry ACN. Dwell time for each transition was 20 ms and inter-channel delay was set to automatic. Instrument operating parameters were as follows: source temperature, 150 °C; capillary voltage, 2.2 kV; desolvation temperature, 450 °C; desolvation gas flow, 700 L h^−1^; cone gas flow, 150 L h^−1^; nebulizer gas flow, 7.0 bar, collision gas flow, 0.15 mL min^−1^. Nitrogen (≥99.995%) was used as cone and desolvation gas, and argon (99.999%) were used as collision gas. Mass spectral fragmentation corresponding to a MRM transition and proposed fragmentation patterns for analytes are available in Supplementary Figs S13 and S14, respectively.

**Table 1:** Optimized MRM conditions and product ions.

Compound	Transition for quantification Cone (V)/Collision (eV)	Transition for confirmation Cone (V)/Collision (eV)	Reference
Oleacein (**3**)	319.2 > 195.0 [M-H-C_7_H_8_O_2_]^−^	319.2 > 165.0 [M-H-C_8_H_10_O_3_]^−^	^6, 22, 23^
	8/6	8/8	
Oleocanthal (**4**)	303.2 > 179.0 [M-H-C_7_H_8_O_2_]^−^	303.2 > 165.0 [M-H-C_8_H_10_O_2_]^−^	^6, 22, 23^
	12/6	12/10	
Oleuropein aglycone (**5**)	377.3 > 275.1 [M-H-C_4_H_6_O_3_]^−^	377.3 > 307.1 [M-H-C_4_H_6_O]^−^	^6, 8^
	8/14 [M-H-C_5_H_10_O_2_]^−^	8/10	
Ligstroside aglycone (**6**)	361.3 > 291.1 [M-H-C_4_H_6_O]^−^	361.3> 259.1 [M-H-C_4_H_6_O_3_]^−^	^6, 8^
	36/10	36/10 [M-H-C_5_H_10_O_2_]^−^	
Hydroxytyrosol-d_5_ (**1-d**_**5**_)	158.0 > 126.0 [M-H-CD_2_O]	158.0 > 97.9	^35^
	10/14	10/20	

## Results

### Detection of analytes and chromatographic separation

Owing to keto-enol tautomerism, which involves opening the secoiridoid ring, monoaldehydic and dialdehydic forms of the aglycones are in equilibrium^47^. Monoaldehydic forms are favoured in the olive oil, although in specific varieties and also depending on the oil production parameters, dialdehydic forms can also be major phenolics^10^. For each aglycone, both aldehydic forms share almost all transitions from precursor/parent ion to product/daughter ions in negative ESI, as well as the occurrence between all transitions is very similar^6,8,25^. MRM transitions in aglycones were selected in order to quantify and confirm with two transitions all the isomers of each aglycone.

On the other hand, oxygenated oleocanthal (oleocanthalic acid^48^) and oxygenated ligstroside aglycone have the same molecular formula and precursor ion as oleacein and oleuropein aglycone, respectively, but their fragmentation originates different daughter ions^6^. Taxifolin, a phenolic compound of the flavonoid family with the same molecular weight as oleocanthal, can be found in olive oils at <1 mg Kg^−1^ ^49^. With the analytical method proposed, detecting the taxifolin is almost impossible due to the high dilution of the sample. In addition, the mechanism of fragmentation of taxifolin^50^ and oleocanthal in negative ESI are different and do not share daughter ions.

**Figure 3:**
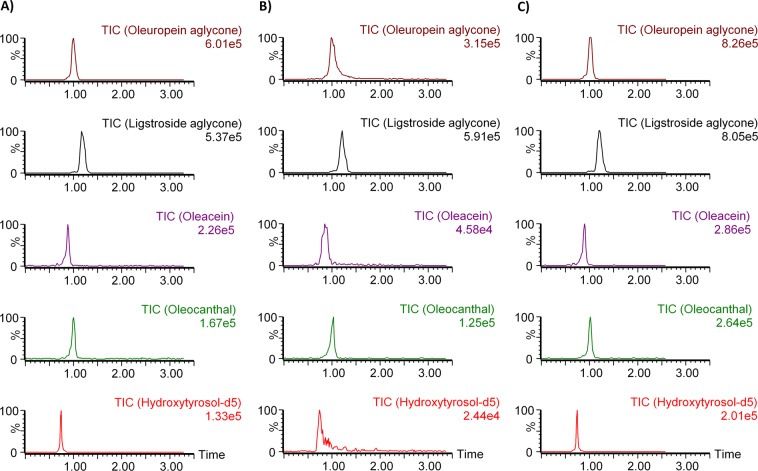
Chromatographic separation using as mobile phase A: water with 0.1% formic acid (**A**), water with 0.05% ammonium (**B**) and water wihout additive (**C**).

Methanol or acetonitrile are the organic solvents commonly used as mobile phase B in the determination of phenols by LC/MS. According to Bajoub *et al*.^51^, the artificial formation of some secoiridoid derivatives happens as long as methanol (and probably water and/or their mixtures) is involved in the sample preparation or has any interaction at any point of the analytical procedure with the analytes of this paper. Therefore, to avoid the formation of these “artificial isomers”, in addition to the direct injection into UHPLC-MS/MS of samples diluted in THF and ACN, as mobile phase B was chosen ACN although the interaction of the aglycones with the silica-based stationary phase or with ACN-H_2_O mixtures gives rise to the isomer (5S, 8S, 9S) of each aglycone^17^. With isocratic elution, the retention time of each analyte is small (<2 min). Then, water with and without additive (0.1% formic acid and 0.05% ammonium) were tested as mobile phase A to maximize sensitivity. As seen in a standard solution of analytes and IS, the best results were obtained without additive in the water (Fig. 3).

In the course of the chromatographic separation, the formation of methyl hemiacetals and dimethyl acetals of analytes is discarded since MeOH is not used, but the formation of aldehyde hydrates may happen. Karkoula *et al*.^52^ showed that the proportion between hydrated and aldehydic forms depends on the residence time of the analytes in H_2_O/ACN mixtures and Sánchez de Medina *et al*.^19^ did not detect hydrated forms of oleacein and oleocanthal within 4 minutes of chromatographic separation. Therefore, since no water is used at any step of the proposed analytical method prior to chromatography (not even in the preparation of the chromatographic vial) and the separation time is <2 min, the posible formation of monohydrates is minimized to the maximum. Nevertheless, according to Celano *et al*., the hydrated forms lose water and a proton in negative ESI, so ions selected by the mass analyzer are the same for the hydrated and non-hydrated forms.

On the other hand, special attention was paid to cleaning of the column after the isocratic separation. Olmo-García *et al*.^28^ establishes a procedure for column cleaning with THF, ACN and isopropanol every 48 injections of sample which are diluted only 5 times. In this work, the samples are diluted 500–8000 times (see section 3.3). ACN-THF mixtures have been widely used in the separation of triglycerides by chromatography^53^ due to the high eluent strength of THF. To ensure optimal cleaning of the column, HPLC grade THF for 0.4 min was included in the chromatographic separation and 10 μL of ACN was injected every 15 sample injections. If THF is not included in the chromatography, signal of the analytes is observed in an ACN injection after a sample (Fig. S15).

### Analytical method validation

Calibration curves were obtained in sunflower oil free of the analytes. Sunflower oil samples were diluted and spiked as described in Fig. 2. The aliquot, V_a_, was 100 μL and in the last step, calibration solutions, which contained IS and analytes, were used instead of the IS solution. The calibration was carried out by weighted least squares methods using weight as the inverse of the concentration.

Two chromatography vials for each concentration level (seven level) were done and analysed twice. Relative area (analyte area/internal standard area), used as the analytical signal, was represented versus analyte concentration in the chromatography vial. The results of the calibration for each compound are shown in Table 2.

**Table 2:** Analytical and statistical parameters.

	3	4	5	6
n	28	28	28	28
b	3.7·10^−2^	5.0·10^−3^	4.0·10^−2^	2.7·10^−2^
S_b_	7.2·10^−4^	1.1·10^−5^	4.9·10^−4^	5.3·10^−4^
S_y/x_	1.2·10^−2^	6.2·10^−3^	3.5·10^−3^	9.5·10^−3^
R^2^ (%)	99.5	99.4	99.6	99.1
P_DW_ (%)	10.1	7.2	96.0	15.3
LOD (ng mL^−1^)	1.0	4	0.3	1.0
LOQ (ng mL^−1^)	3.3	12	0.9	3.5
LDR (ng mL^−1^)	3.3–220	12–792	0.9–100	3.5–208

n: points of calibration; b: slope; S_b_: slope standard deviation; S_y/x_: regression standard deviation; R^2^: determination coefficient; %P_DW_: %P value of Durbin–Watson statistic; LOD: limit of detection; LOQ: limit of quantification; LDR: linear dynamic range.

Linearity, sensitivity and accuracy (trueness and precision) of the analytical method were evaluated according to the US Food and Drugs Administration (FDA) guideline for Bioanalytical Method Validation^54^.

#### Linearity

The linear correlation coefficient (R^2^) in all cases was greater than 99%, which indicates good linearity within the established ranges. No autocorrelation was observed in the residuals of the linear regression since all P values of Durbin–Watson statistic were >5%. Table 2 shows these data.

#### Sensitivity

The limit of detection (LOD) and the limit of quantification (LOQ) were calculated as N·S_y/x_/b. The term N is 3 and 10 for LOD and LOQ, respectively. The results are shown in Table 2.

#### Accuracy (precision and trueness)

To evaluate the accuracy, sunflower oil samples spiked at four concentration levels for each compound were prepared as described in the calibration. Five chromatography vials for each concentration level were done in the same day and analysed twice. The relative standard deviation (% RSD) and the relative error (% RE) were calculated for each concentration level.

As shown in Table 3, the RSD and RE values were <15% in all cases, which are in compliance with the FDA guidelines. The values obtained indicate that this method is accurate.

**Table 3:** Matrix effect and accuracy of the analytical method.

	^a^Spiked (ng mL^−1^)	^b^RE (%)	^c^RSD (%)	^d^ME (%)
**3**	6.9	6.7	2.1	13.7
	27.6	10.0	2.2	3.7
	165.6	4.6	0.8	6.4
	220.8	4.0	2.4	—
**4**	24.75	6.1	8.5	8.6
	99	1.5	2.9	1.1
	594	6.3	2.5	14.1
	792	4.1	3.8	—
**5**	3.125	7.4	3.7	4.2
	12.5	7.7	1.5	7.3
	75	4.7	1.2	10.6
	100	2.1	3.5	—
**6**	6.5	9.8	3.9	15.8
	26	1.5	3.8	8.8
	156	10.5	1.8	1.4
	208	1.9	4.2	—

^a^Concentration in the chromatography vial.

^b^R (%): recovery (mean of ten determinations).

^c^RSD (%): relative standard deviation (mean of ten determinations).

^d^ME (%): matrix effect (mean of four determinations).

#### Matrix effect

Matrix effect (ME) was evaluated, for each analyte, by comparing its relative area in sunflower oil (*A*_*SO*_) to that of a olive oil sample spiked (*A*_*OO*_) at the same concentration. Spiked procedure of both oils was the same as that used in the calibration. Three concentration levels were evaluated and for each of them, two aliquots were prepared and analyzed twice. Due to the difficulty of finding olive oil suitable for human consumption and free of phenolic secoiridoids, the analytical signal produced by the olive oil sample (*B*_*OO*_) was subtracted from each aliquot of spiked olive oil. The matrix effect was calculated by Eq. (1).

1 $$ME( \% )=\frac{{A}_{SO}-({A}_{OO}-\,{B}_{OO})mL}{{A}_{SO}}\times 100$$

All the values of the matrix effect (see Table 3), except one, were less than 15% and close to the RSD values of the analytical method. Therefore, it can be affirmed that the matrix effect is not significant.

### Application of the method

The method of analysis was applied to the six samples collected. The concentration of each secoiridoid was expressed as mg kg^−1^ using Eq. (2). For each olive oil sample, two chromatography vials were made and each one analysed twice. An aliquot of 100 μL (N = 20) was used in all samples but for some EVOOs, 25 μL (N = 80) and/or 400 μL (N = 5) were also used. The results are in Table 4.

2 $$C(\frac{mg}{kg})=C(\frac{ng}{mL})\cdot 10\cdot N\cdot 10\frac{mL}{g}\cdot {10}^{-3}\frac{\mu g}{ng}$$

**Table 4:** Method application to commercial EVOO samples.

	O1	O2	O3	O4	O5	O6
^**a**^ **Concentration (mg kg** ^**−1**^ **) (SD)**						
**3**	104 (7)	225 (15)	69 (2)	92 (5)	120 (10)	115 (5)
**4**	118 (8)	160 (10)	180 (7)	115 (9)	99 (4)	104 (6)
**5**	270 (20)	69 (6)	38 (2)	141 (8)	300 (25)	200 (15)
**6**	515 (20)	71 (6)	108 (8)	255 (20)	445 (25)	295 (20)

^a^Mean of four determination; SD: standard deviation.

The concentration range was 69–225, 99–180, 38–300 and 71–515 mg kg^−1^ for **3**, **4**, **5** and **6**, respectively. Except for EVOOs O2 and O3, the sum of aglycones is greater than the sum of oleacein and oleocanthal. The tyrosol derivatives (**4** and **6**) are more abundant than the hydroxytyrosol derivatives (**3** and **5**) in all cases except one, mainly because ligstroside aglycone is the main secoiridoid in four samples. The total content of secoiridoids, whose range was from 376 to 1007 mg kg^−1^, in all the EVOOs analysed exceeds the minimum value (250 mg kg^−1^) to be able to include the health claim for olive oil polyphenols in their labelling. This method is therefore a good alternative for the selective quantification of secoiridoids, a step beyond the classical not specific quantification.

**Figure 4:**
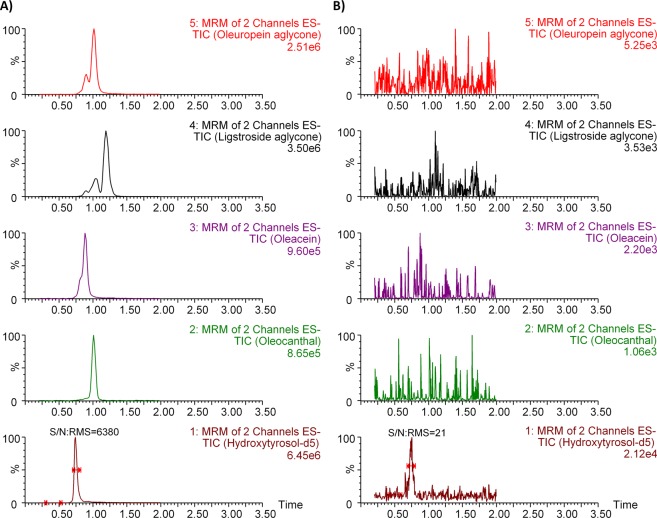
Chromatograms of the sample O3 (**A**) and ACN after several sample injections (**B**).

The chromatogram of sample O3 is shown in Fig. 4A. As shown in Fig. 4B, which corresponds to an ACN injection (10 μL) after several sample injections, the cleaning of the column is very effective since no compound is detected (except <1% of the internal standard). In all samples, there are one or two more peaks in each algycone compared to the standard solution (Fig. 3C). Probably, these peaks correspond to dialdehydic forms of the aglycones.

## Conclusions

A simple, fast and accurate analytical method for the determination of phenolic secoiridoids in olive oil by UHPLC-MS/MS was developed and validated in this work. The main difference with respect to other chromatographic methods collected in the literature is the joint use of analytical standards and the direct determination of secoiridoids. In fact, it is the first chromatographic analytical method with these two characteristics. Using surrogates is convenient in any analytical method that involves sample extraction. A stable-isotope-labeled (SIL) analogue of the analyte is the most appropriate surrogate in a quantitative analysis by LC-MS and since these are very scarce for phenolic secoiridoids^16^, the method described in this publication is highly recommended. Additionally, LC-MS determination of phenolic secoiridoids with pure standards of all the analytes is the most reliable and accurate situation^55^. Tandem mass spectrometry (MS/MS), due to its high sensitivity and selectivity compared to other techniques such as NMR, can be applied to evaluate the presence of virgin or extra virgin olive oil as additive or ingredient in food by determination of the secoiridoids studied in this work.

Owing to the lowest cost of the analytical method compared to the published NMR methods and their short analysis time, the proposed method can be implemented in large olive oil mills as a routine method to evaluate the health claim. On the other hand, this method can be applied to observe the evolution of oleacein, olecocanthal, oleuropein aglycone and ligstroside aglycone since they can undergo hydrolysis and oxidation during storage of olive oil^56,57^.

For these reasons, we consider that the proposed analytical method provides the olive oil sector with a powerful analytical tool that can be used, together with the rest of analytical methods included in EEC regulation 2568/91, to determine the unique characteristics of olive oil.

## Supplementary information

Supplementary information
