# Supplementary material for: Direct determination of phenolic secoiridoids in olive oil by ultra-high performance liquid chromatography-triple quadruple mass spectrometry analysis
Source: Sci Rep. 2019 Oct 29;9:15545. doi: 10.1038/s41598-019-52060-5 (PMC6821043; doi:10.1038/s41598-019-52060-5)
Supplement: Supplementary file 1 — Supplementary information [file 41598_2019_52060_MOESM1_ESM.pdf]

SUPPLEMENTARY INFORMATION

Direct determination of four phenolic secoiridoids in oil olive by ultra-high performance liquid chromatography-triple quadruple mass spectrometry analysis

A. Luque-Muñoz,[a] R. Tapia,[b] A. Haidour,*,[a] J. Justicia,[b] and J. M. Cuerva*,[b]

[a] *Nuclear Magnetic Resonance Unit, Scientific Instrumentation Center, University of Granada, E-18071 Granada, Spain*

[b] *Department of Organic Chemistry, University of Granada, Campus Fuentenueva s/n, E-18071 Granada, Spain*

* Corresponding authors: jmcuerva@ugr.es (Dr. Juan M. Cuerva), ahaidour@ugr.es (Dr. A. Haidour)

Contents

- Synthesis and purification of analytes S1
- ^{1}H, ^{13}C, 2D-COSY and 2D-HSQC spectra for **3** S2-S3
- ^{1}H, ^{13}C, 2D-COSY and 2D-HSQC spectra for **4** S4-S5
- ^{1}H, ^{13}C, 2D-COSY and 2D-HSQC spectra for **5** S6-S7
- ^{1}H, ^{13}C, 2D-COSY and 2D-HSQC spectra for **6** S8-S9
- ^{1}H, ^{13}C, and 2D-HSQC spectra for **1-d$_5$** S10-S11
- Assignment of ^{1}H NMR spectrum for **3**, **4**, **5** and **6** S12-S13
- H1 and H3 assigned to each diastereomer of **4** and **6** S14
- 2D-HSQC assignment of the elenolic fragment for the diastereomers of **4** and **6** S15
- Mass spectral fragmentation of the $[M{-}H]^-$ ion of **3**, **4**, **5**, **6** and **1-d$_5$** S16-S17
- Proposed fragmentation mechanisms for **3**, **4**, **5** and **6** S18
- Chromatograms of ACN injections after analyzing a sample with absence and presence of TFH in the chromatographic gradient S19
- Chromatographic program of the analytical method S20
- Supplementary references S21

Synthesis and purification of analytes

The synthesis of the analytes was carried out using bibliography:

- Oleacein and oleocanthal were semi-synthesized in one step, using oleuropein and ligstroside as precursors, under Krapcho decarbomethoxylation conditions [1], respectively.
- Monoaldehydic oleuropein aglycone and monoaldehydic ligstroside aglycone were synthesized from oleuropein and ligstroside, respectively, following the procedure described in literature [2].

Oleuropein and ligstroside were extracted from olive leaves, after treatment in boiling water for 1 hour. Then, this aqueous phase was extracted with butanol (x2), dried over anhydrous Na_2SO_4, and the solvent was removed. The residue was purified by flash chromatography in silica gel (CH_2Cl_2: MeOH 8:2), to yield the corresponding oleuropein or ligstroside.

The analytes were purified by preparative HPLC. The column used was a Zorbax Rx-SIL column (5 m; 9.4 × 250 mm), and hexane and ethyl acetate were the solvents.

Figure S1. ^{1}H NMR spectrum (up) and ^{13}C NMR spectrum (down) of oleacein (**3**) in $CDCl_3$. These spectra match those of literature [3–5].

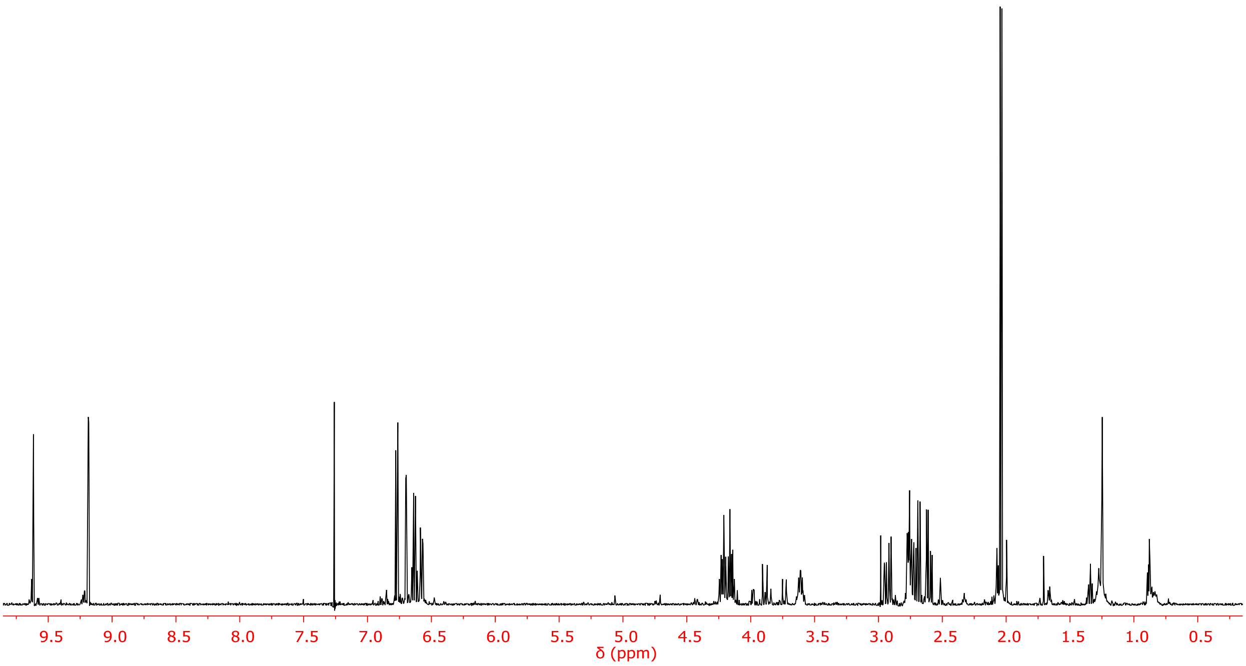

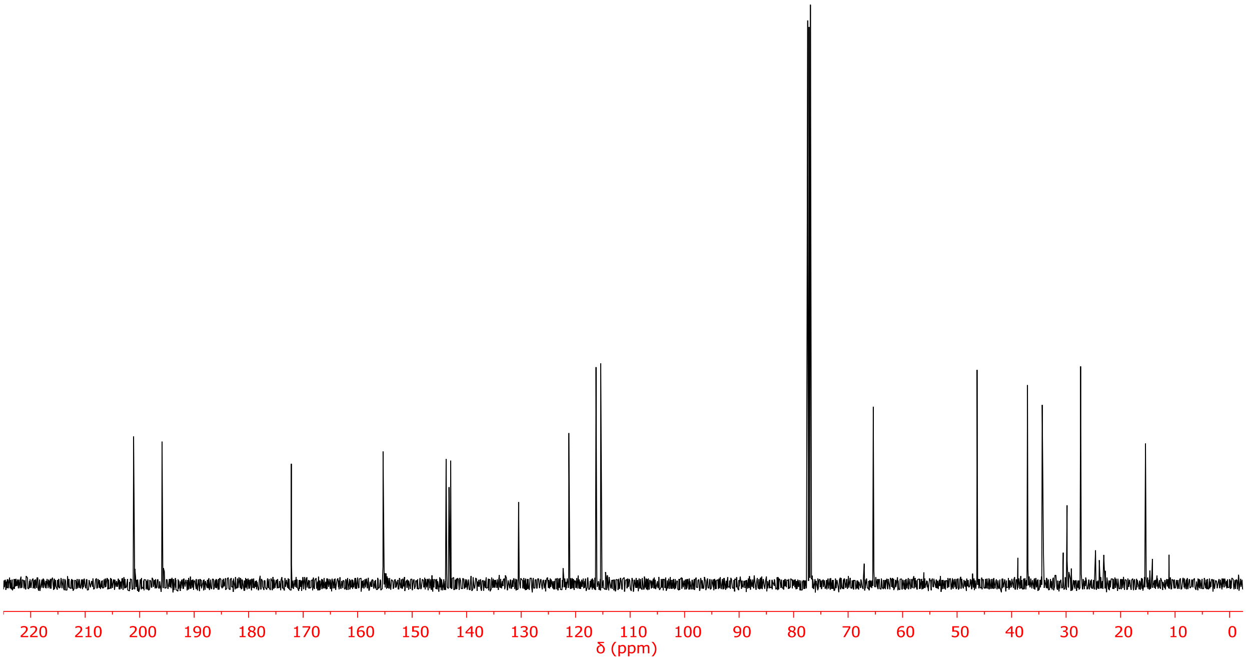

Figure S2. 2D-COSY spectrum (up) and 2D-HSQC spectrum (down) of oleacein (**3**) in $CDCl_3$.

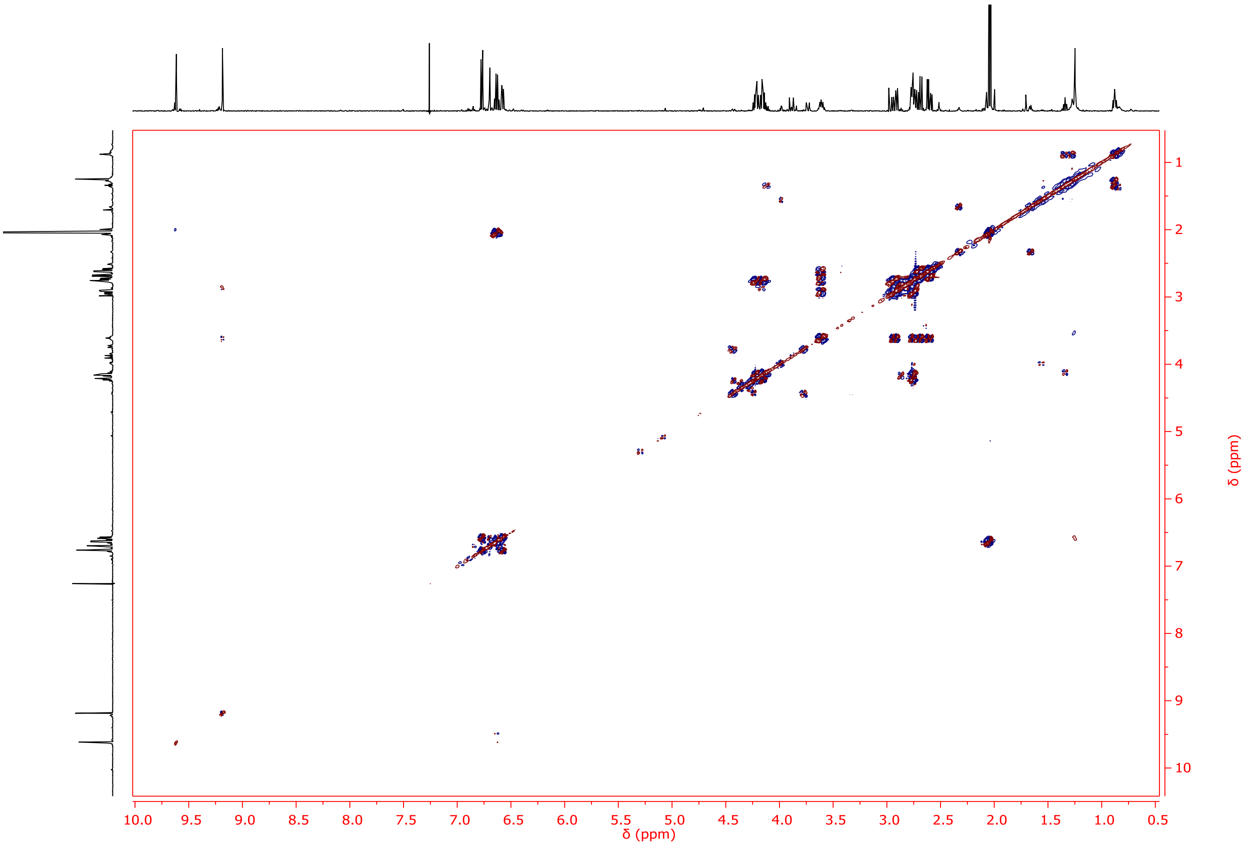

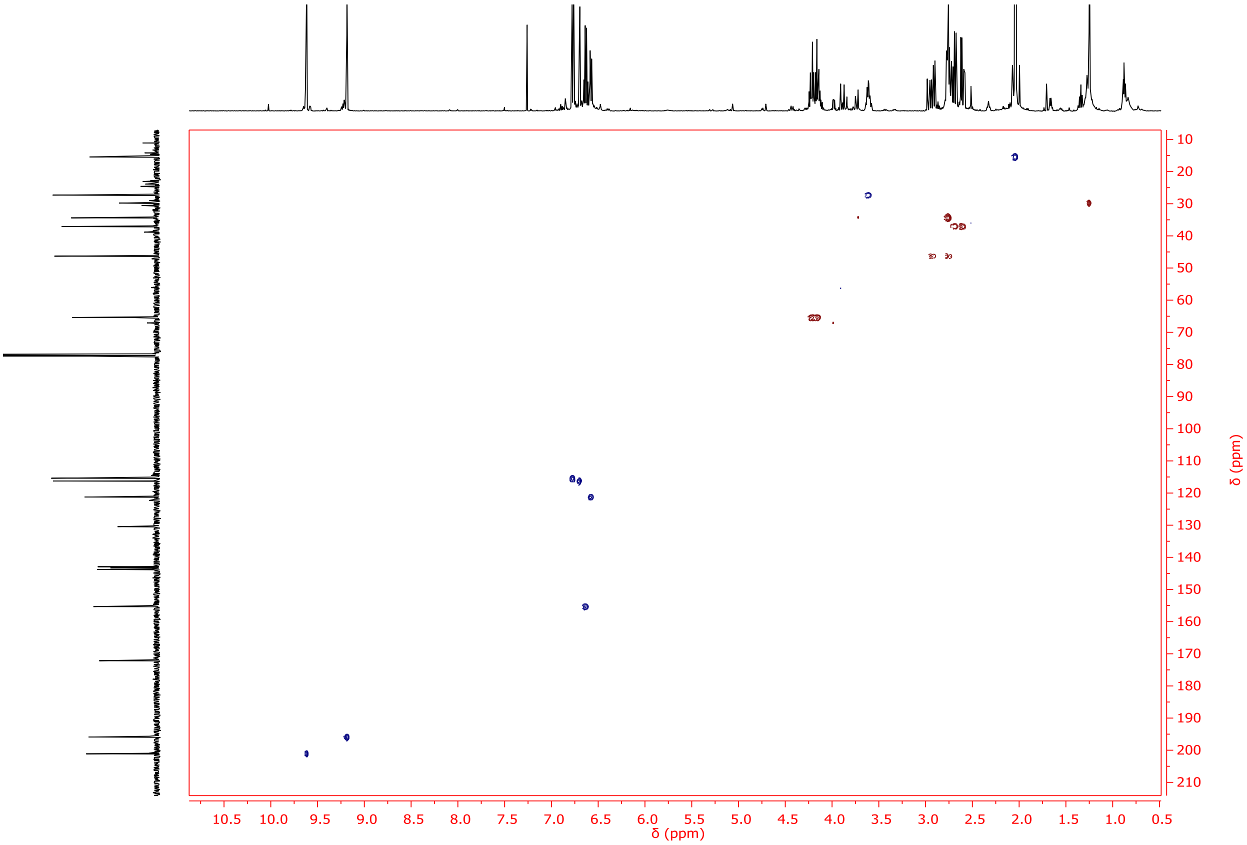

Figure S3. ^{1}H NMR spectrum (up) and ^{13}C NMR spectrum (down) of oleocanthal (**4**) in $CDCl_3$. These spectra match those of literature [3–5].

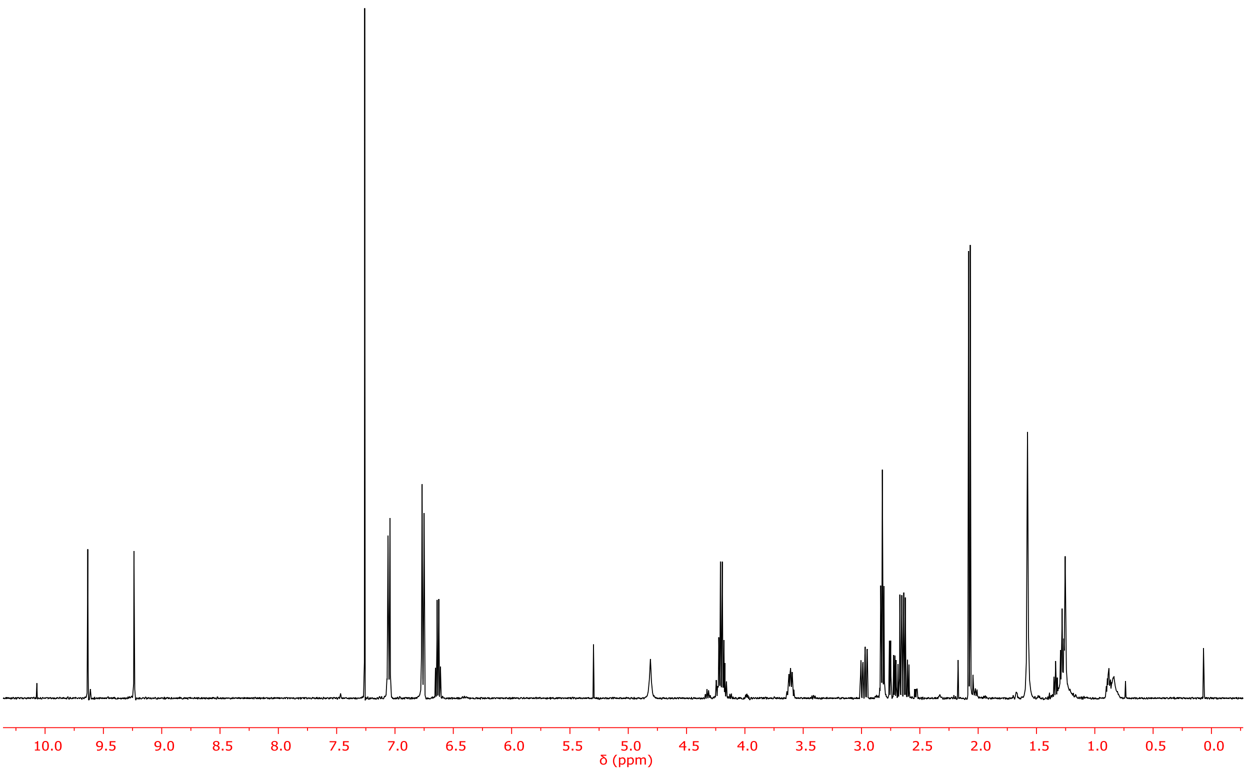

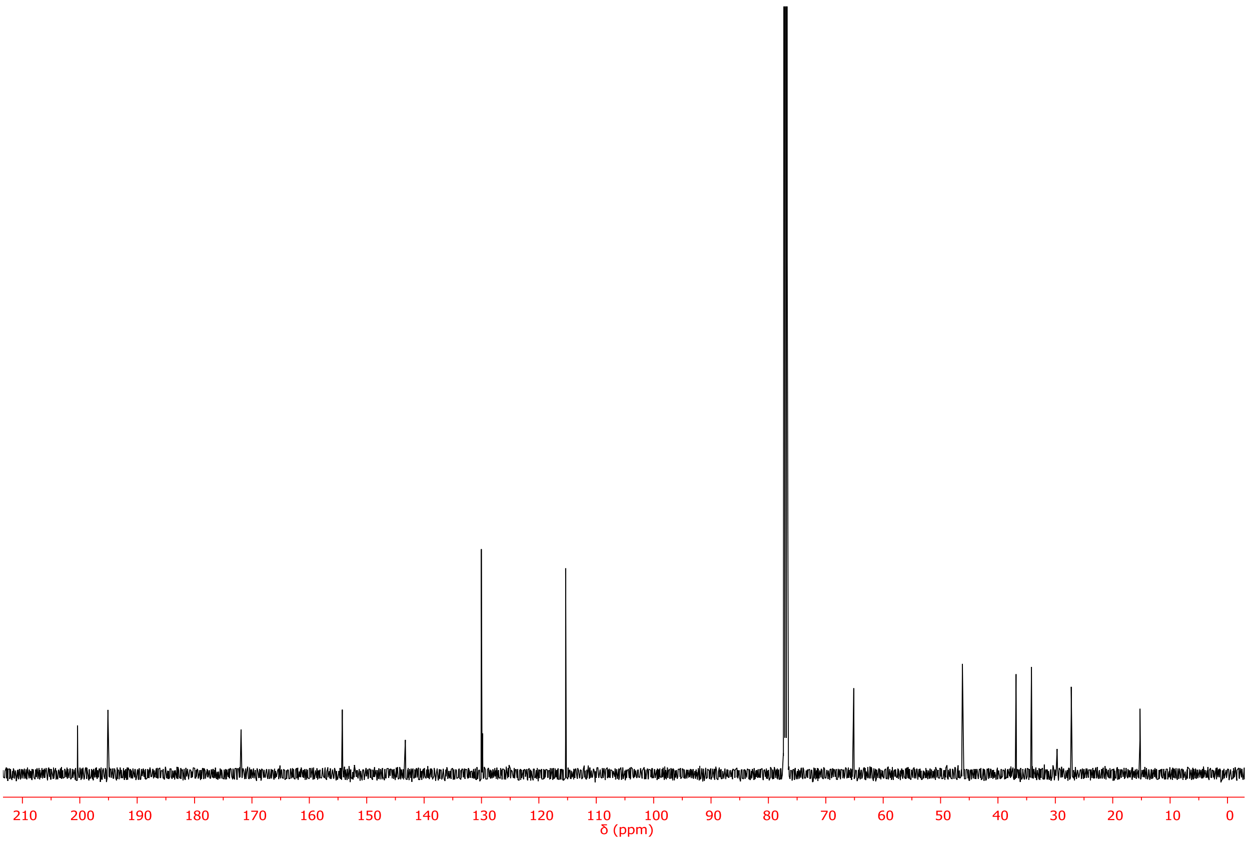

Figure S4. 2D-COSY spectrum (up) and 2D-HSQC spectrum (down) of oleocanthal (**4**) in $CDCl_3$.

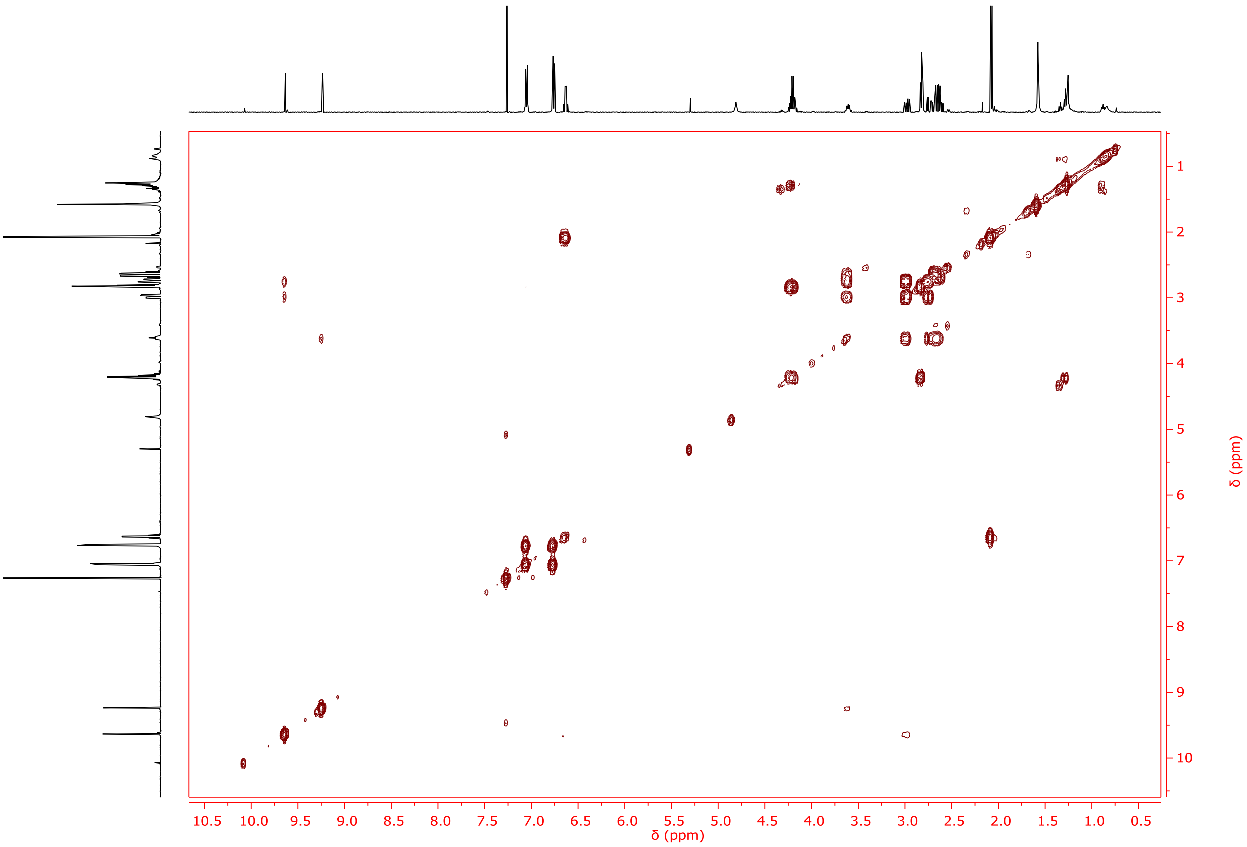

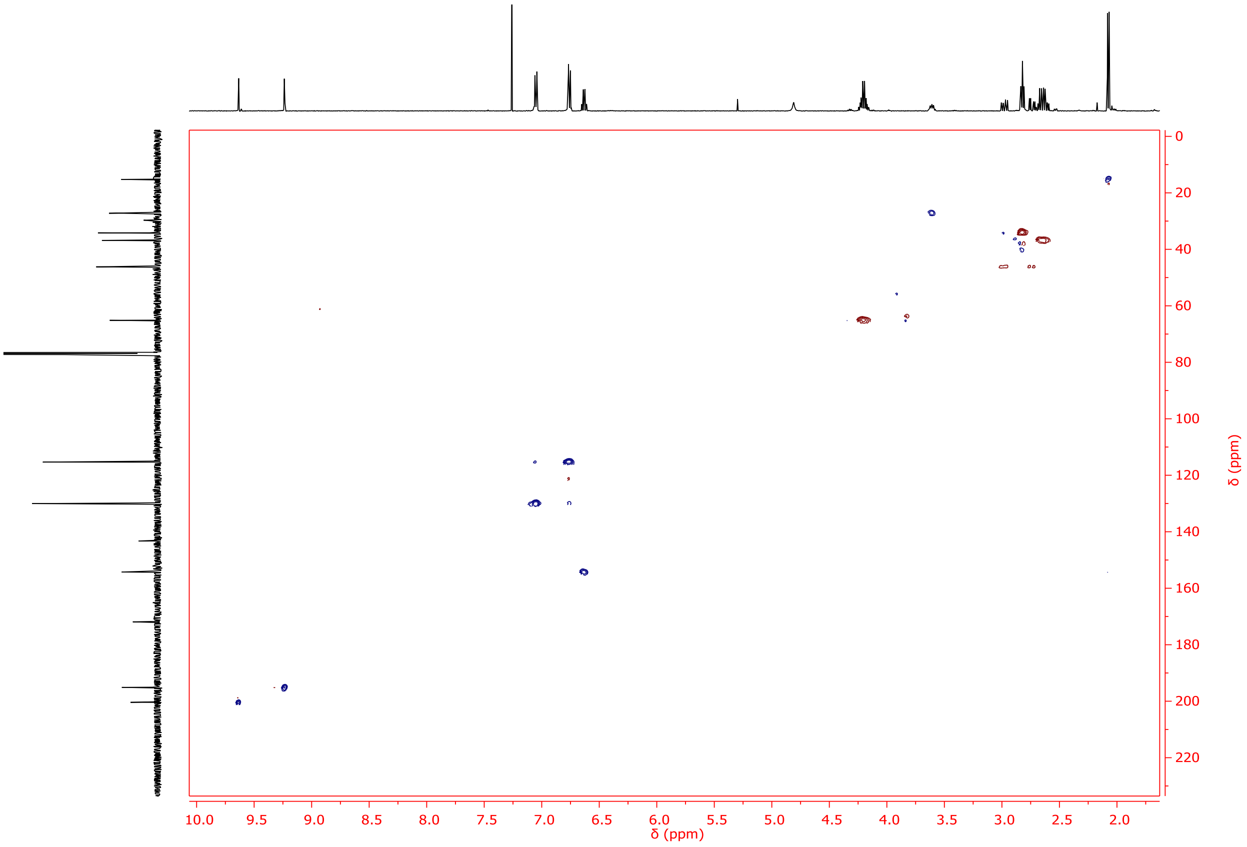

Figure S5. ^{1}H NMR spectrum (up) and ^{13}C NMR spectrum (down) of monoaldehydic oleuropein aglycone (**5**) in $CDCl_3$. ^{1}H and ^{13}C signal assignment has been done with the help of bibliography [3,5–7]. (5S,8S,9S) and (5S,8R,9S) diastereoisomers have already been described in $CDCl_3$ [7].

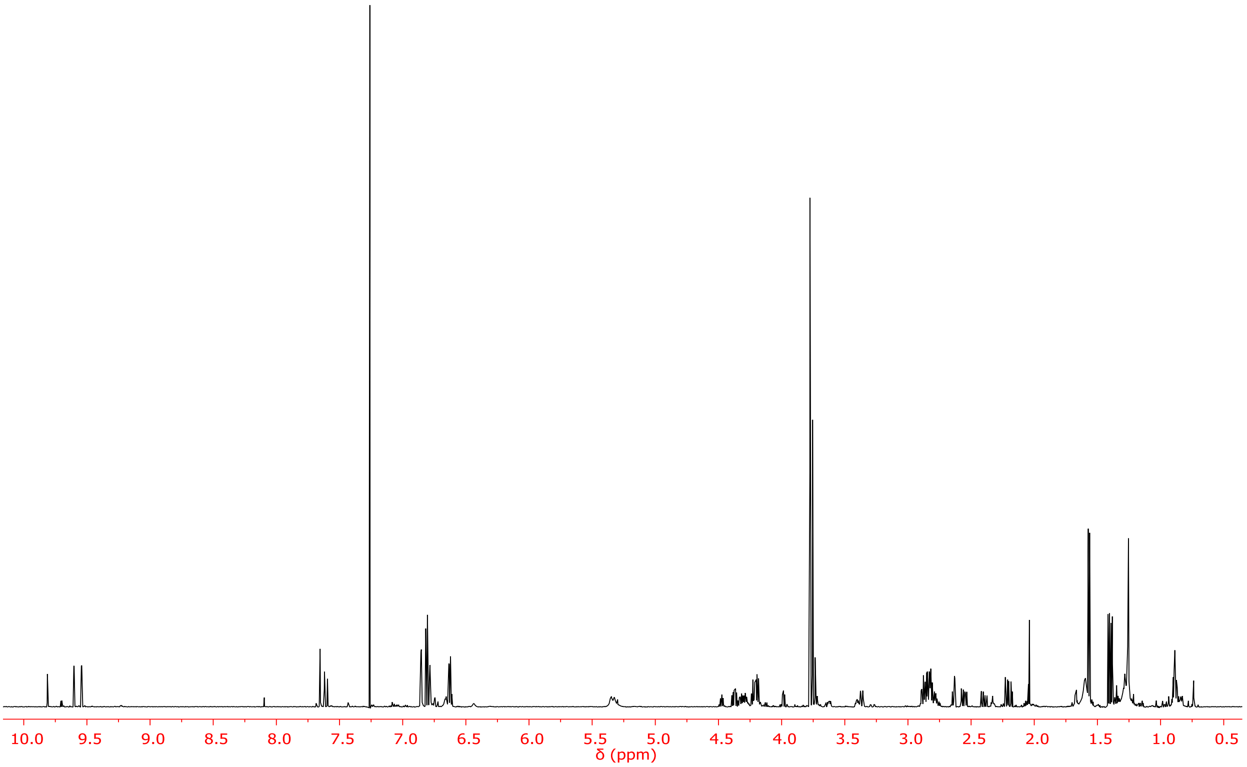

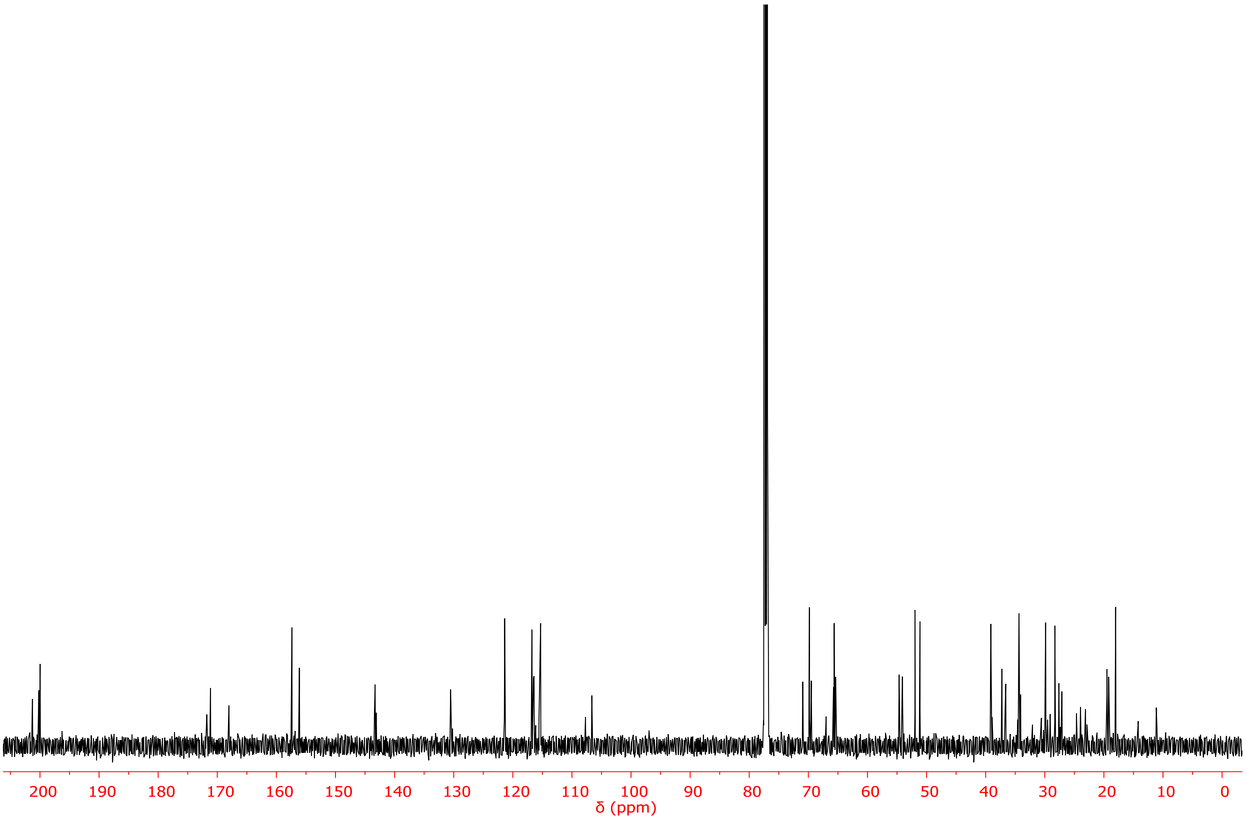

Figure S6. 2D-COSY spectrum (up) and 2D-HSQC spectrum (down) of monoaldehydic oleuropein aglycone (**5**) in $CDCl_3$.

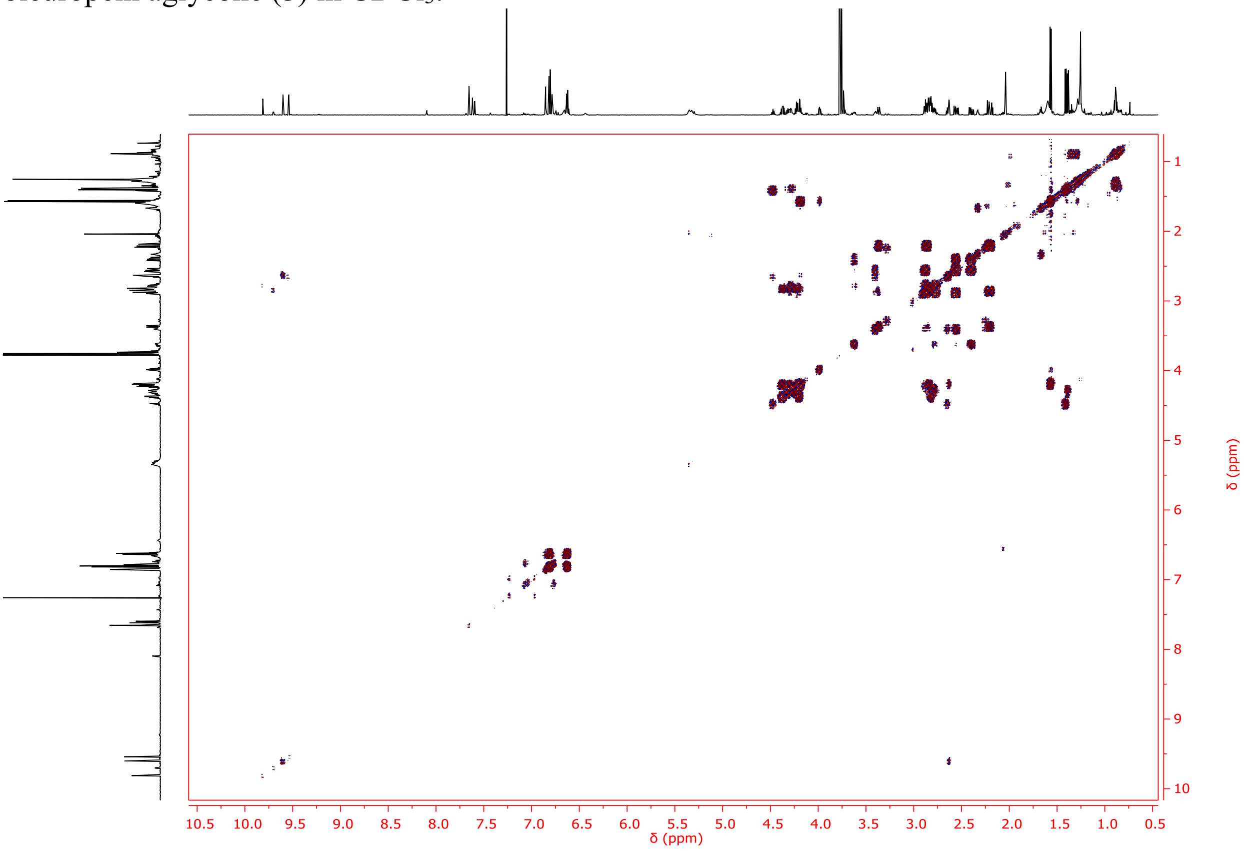

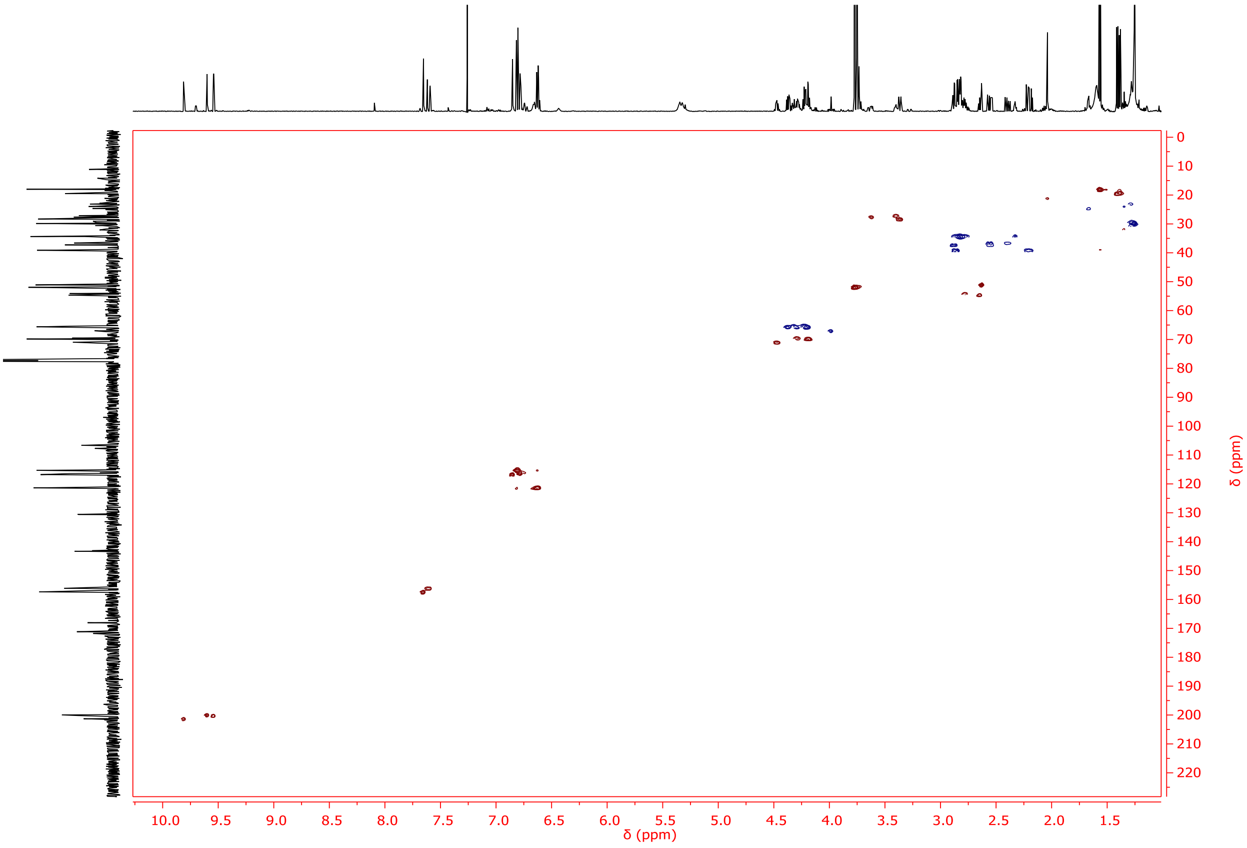

Figure S7. ^{1}H NMR spectrum (up) and ^{13}C NMR spectrum (down) of monoaldehydic ligstroside aglycone (**6**) in $CDCl_3$. ^{1}H and ^{13}C signal assignment has been done with the help of bibliography [6,8]. (5S,8S,9S) diastereoisomers have already been described in $CDCl_3$ [8].

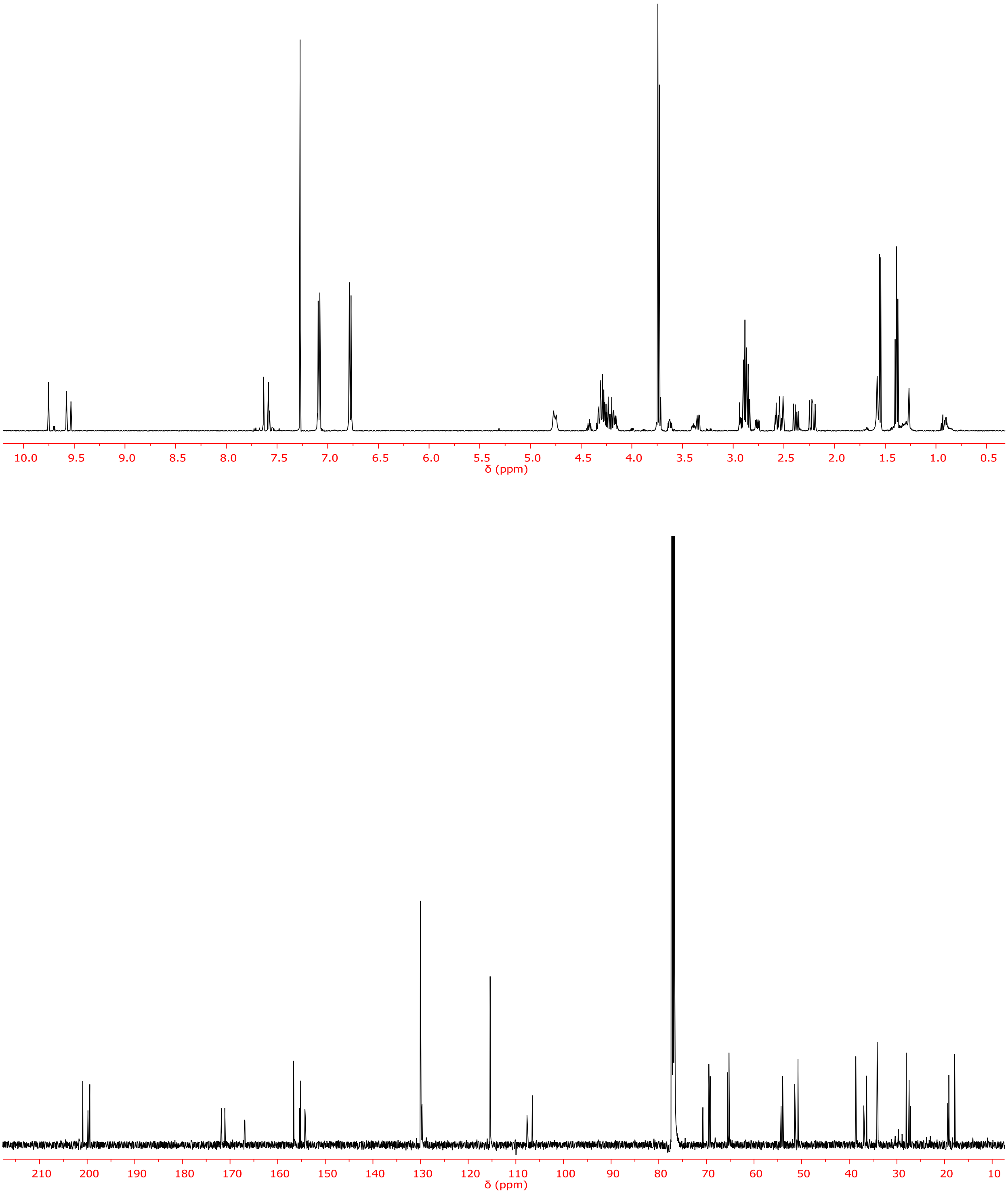

Figure S8. 2D-COSY spectrum (up) and 2D-HSQC spectrum (down) of monoaldehydic ligstroside aglycone (**5**) in $CDCl_3$.

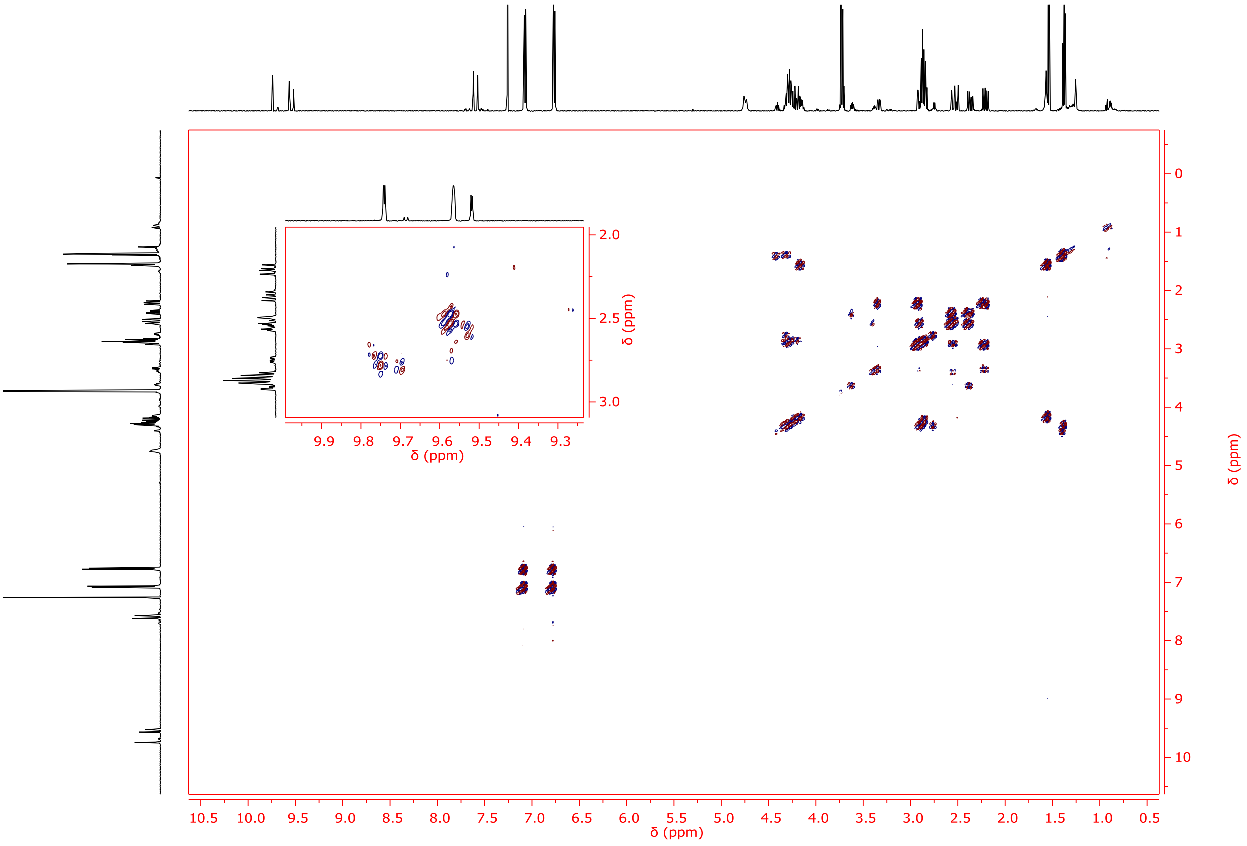

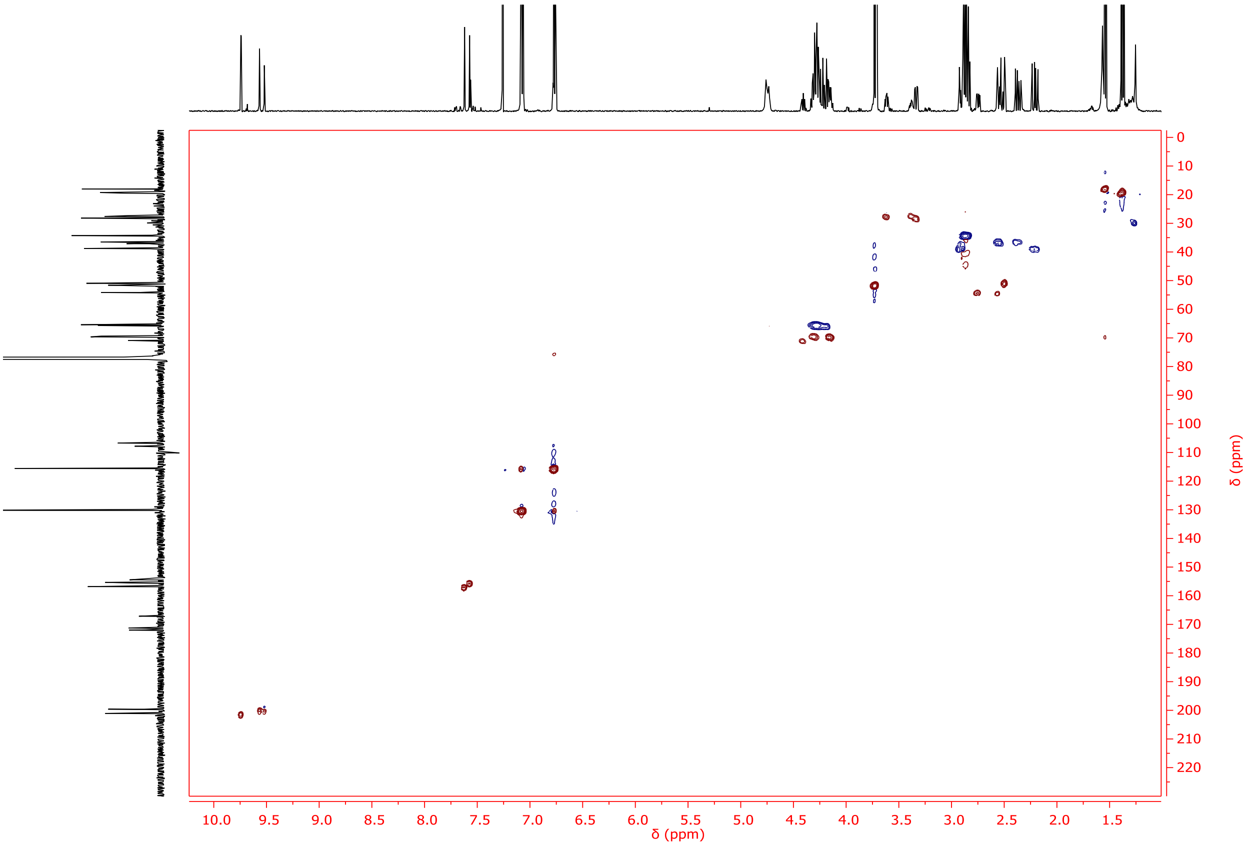

Figure S9. ^{1}H NMR spectrum (up) and ^{13}C NMR spectrum (down) of hydroxytyrosol-d₅ (**1-d₅**) in CD_3OD.

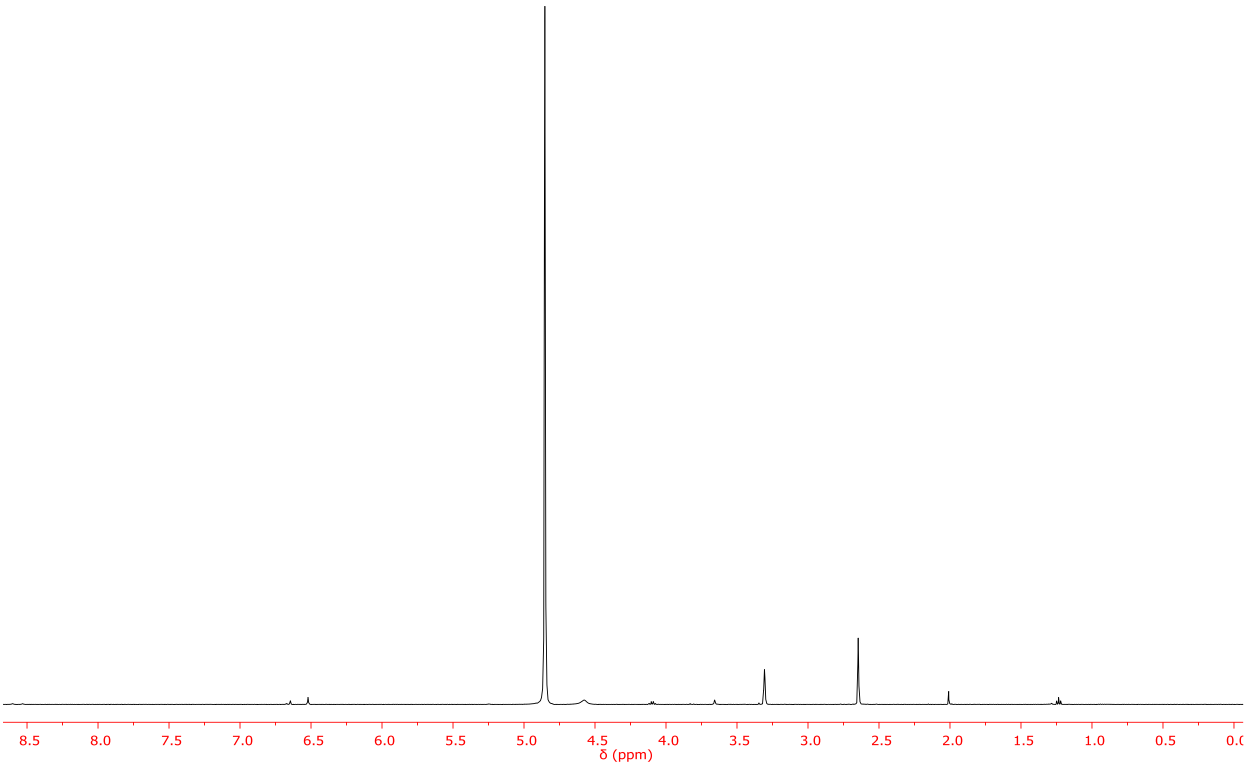

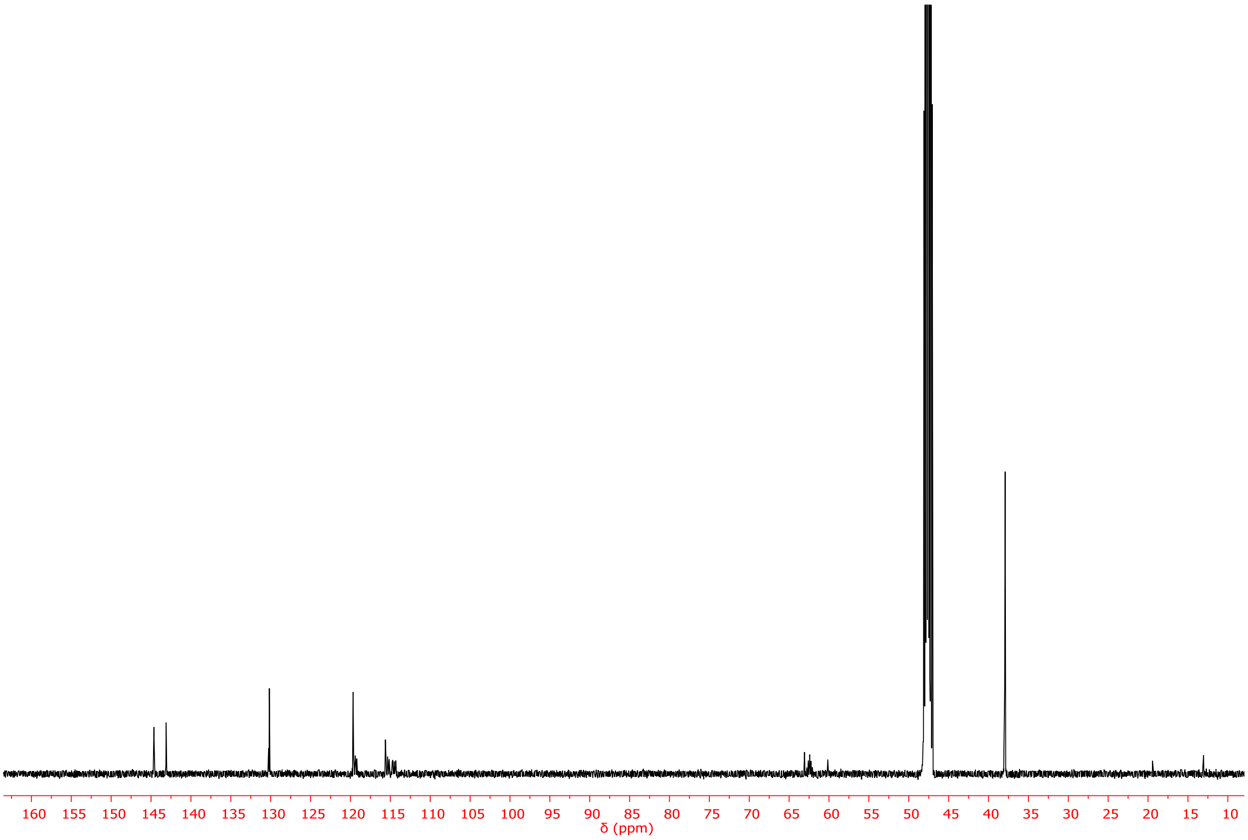

Figure S10. 2D-HSQC spectrum of hydroxytyrosol-d_5 (**1-d$_5$**) in CD_3OD.

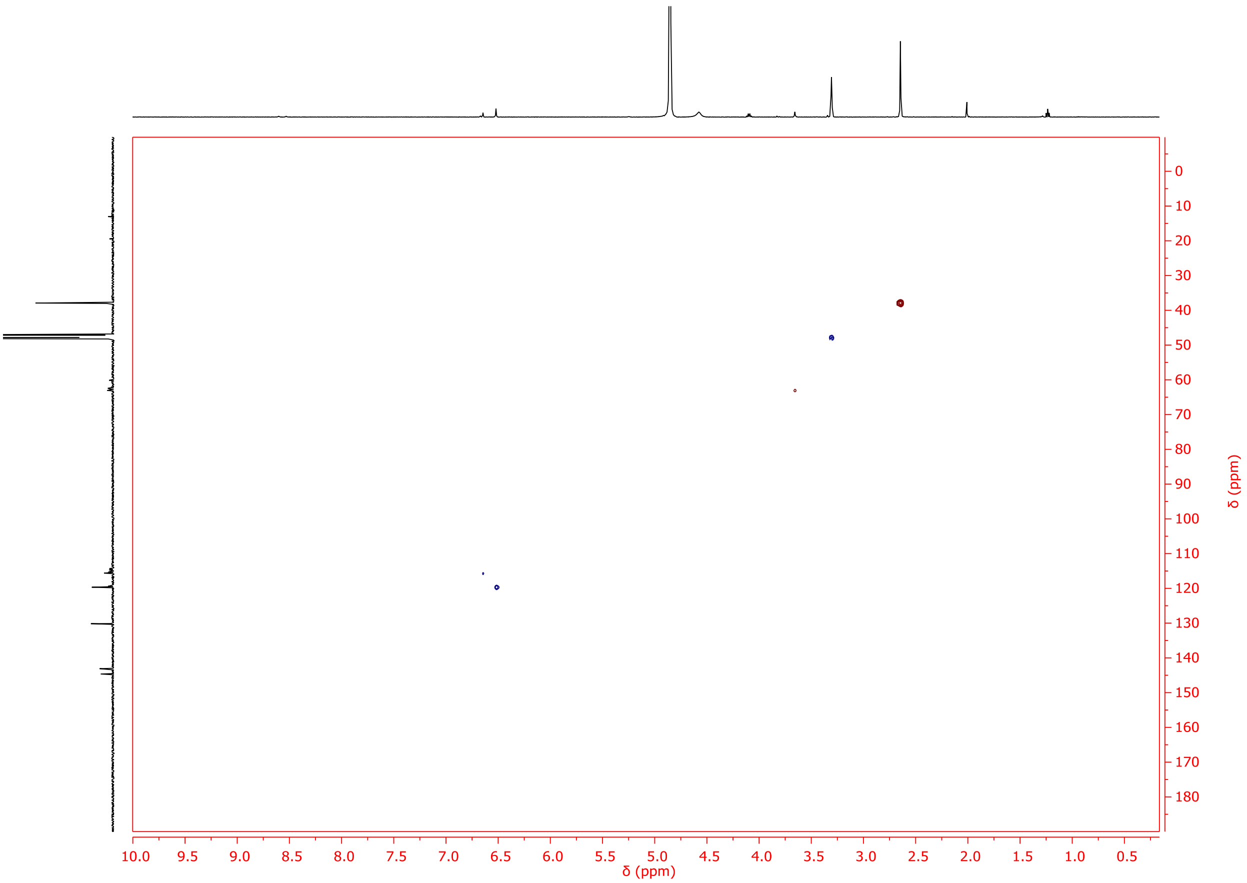

Figure S11. Assignment of ^{1}H NMR spectrum for **3**, **4**, **5** and **6**.

3

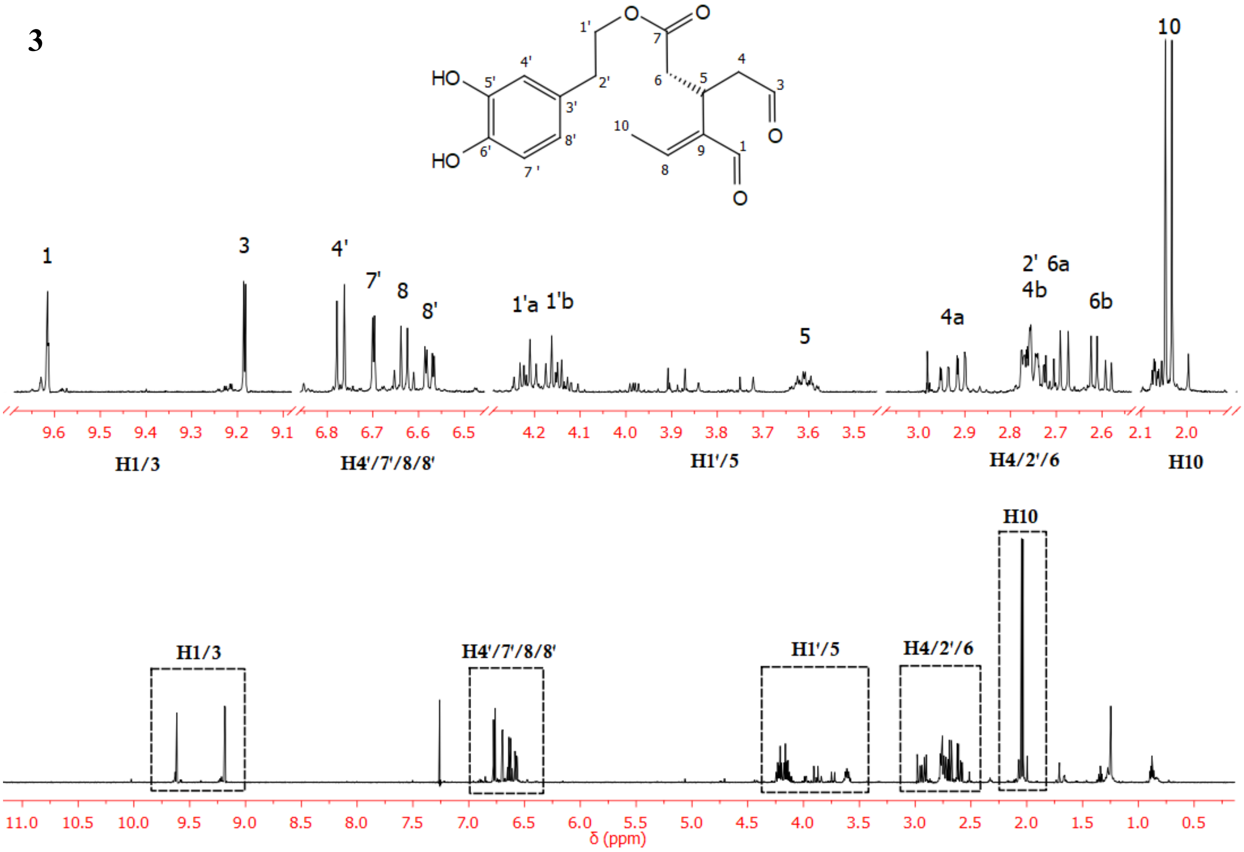

4

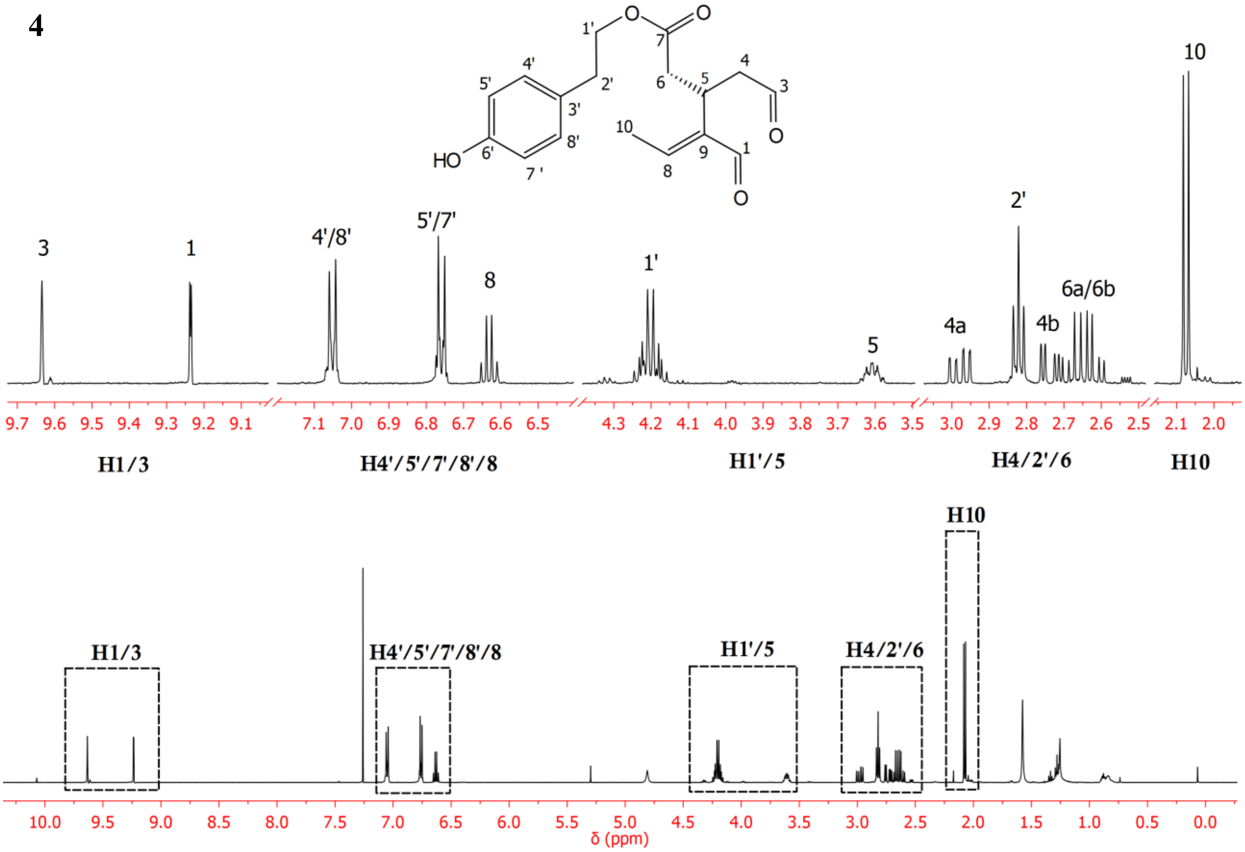

5

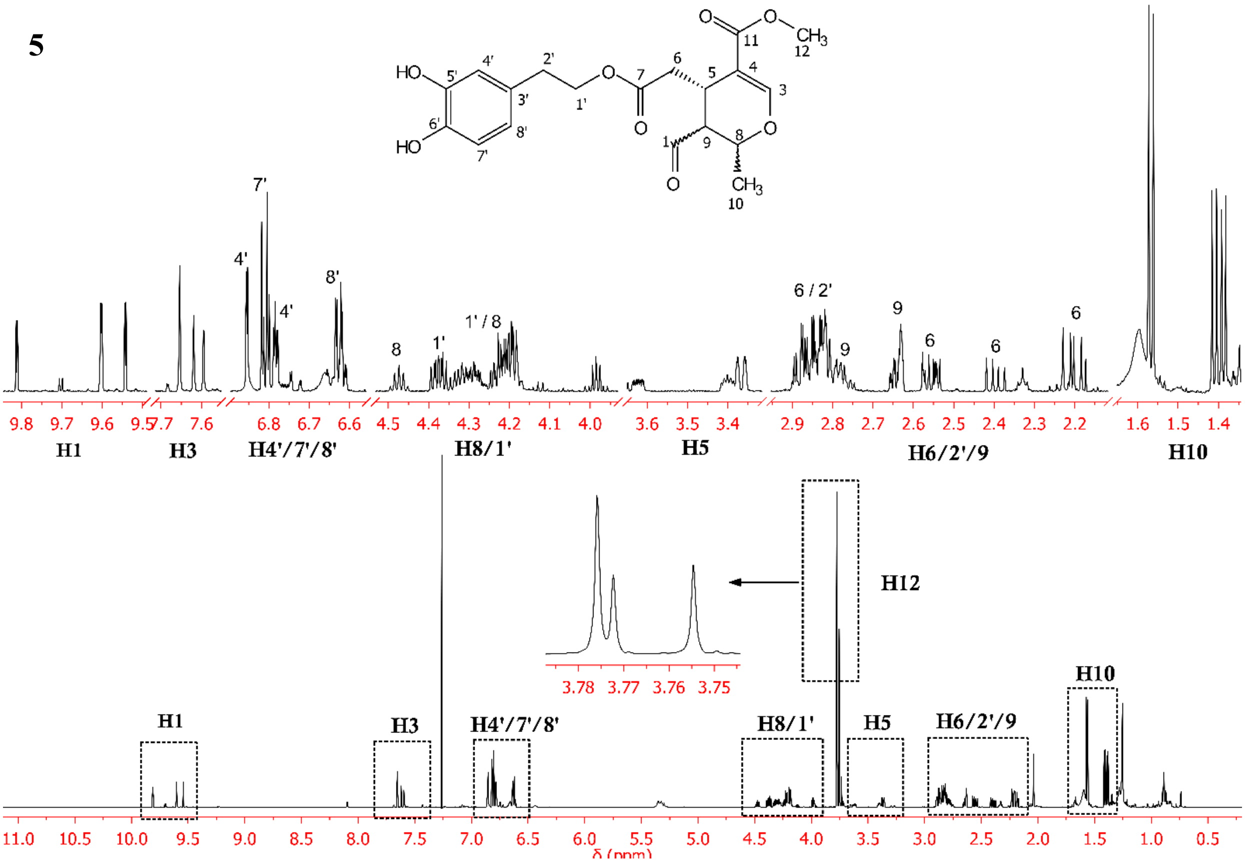
H1
H3
H4'/7'/8'
H8/1'
H5
H6/2'/9
H10
H12
δ (ppm)

6

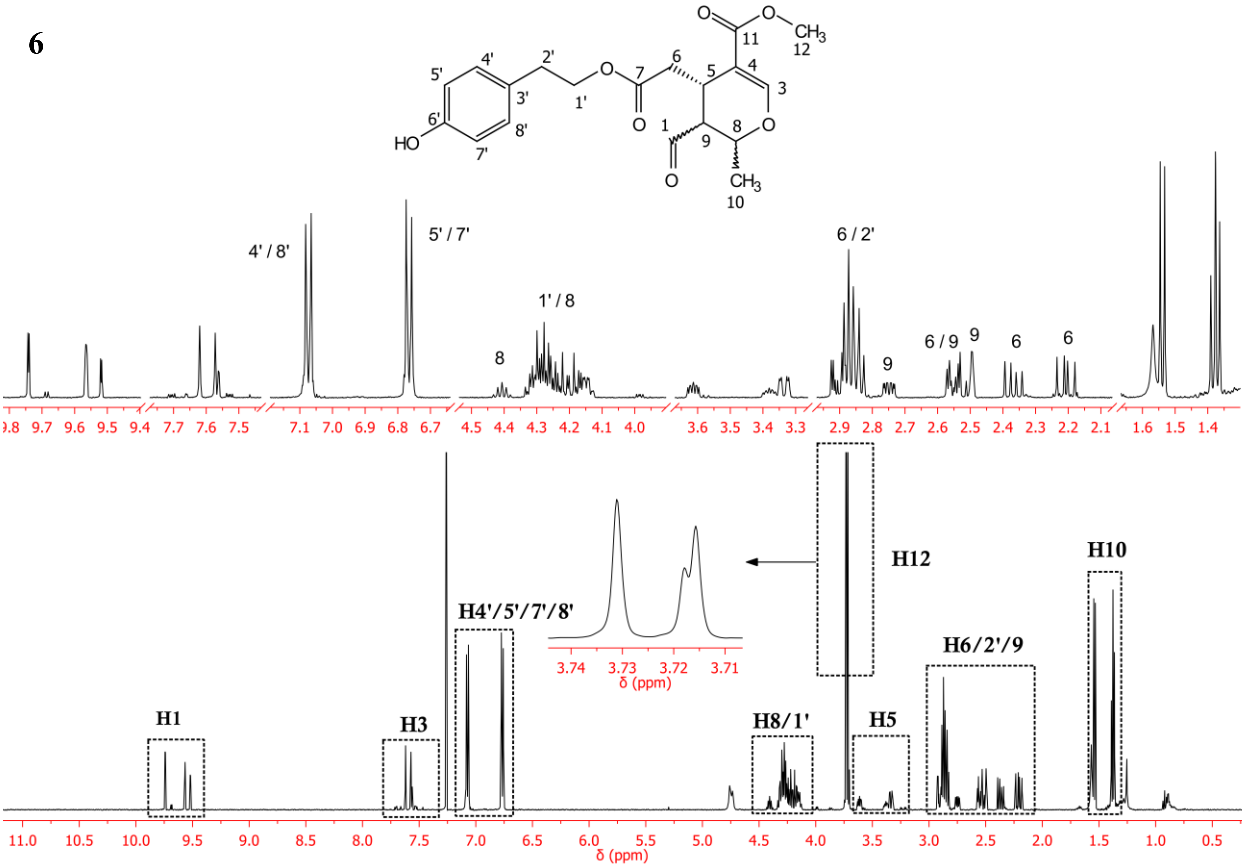
H1
H3
H4'/5'/7'/8'
H8/1'
H5
H6/2'/9
H10
H12
δ (ppm)

Figure S12. H1 and H3 assigned to each diastereomer of a) monoaldehydic oleuropein aglycone and b) monoaldehydic ligstroside aglycone.

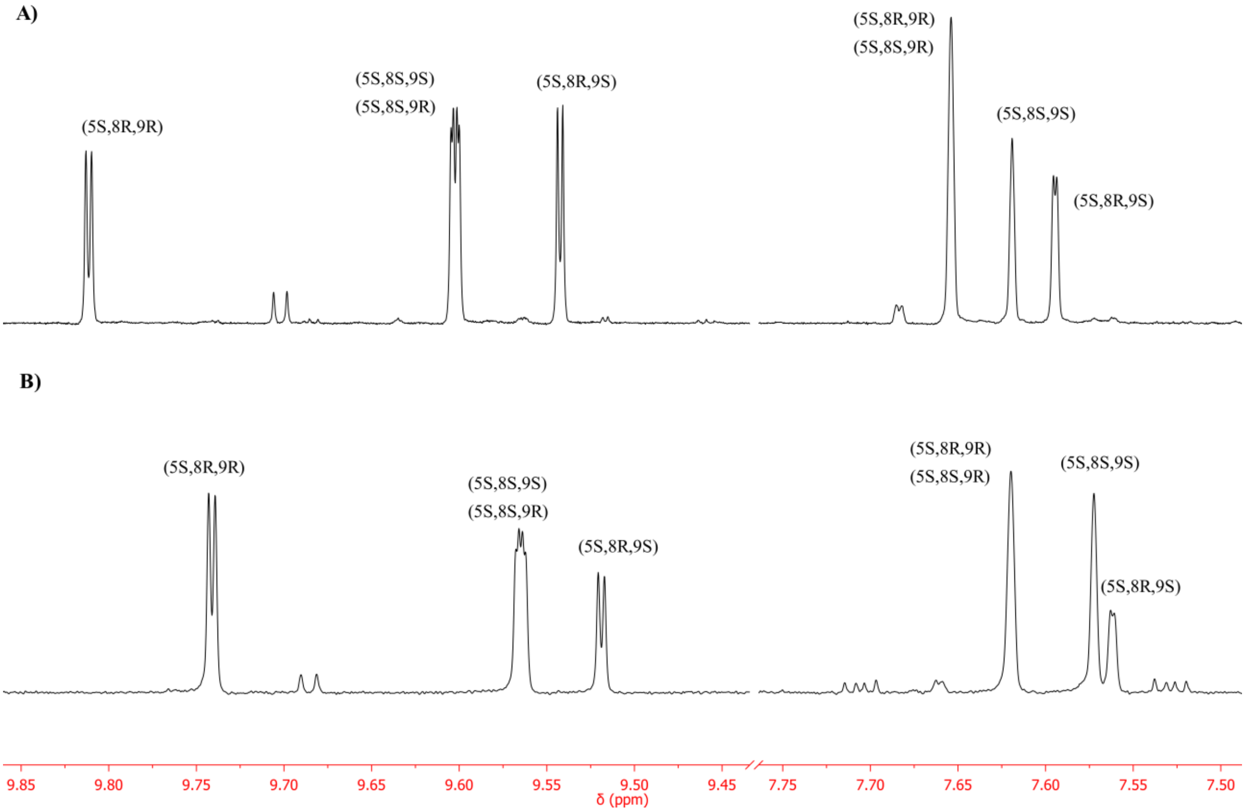

Table S1. 2D-HSQC assignment of the elenolic fragment for the diastereomers of monoaldehydic ligstroside aglycone using 2D-NOESY and 1D-TOCSY. C12 could not be assigned.

	**(5S,8S,9S) / (5S,8S,9R)**	**(5S,8R,9R)**	**(5S,8R,9S)**
1	9.56 – 199.59	9.74 – 201.08	9.52 – 200.00
3	7.62 – 156.82 (8S,9R)<br>7.57 – 155.55 (8S,9S)	7.62 – 156.82	7.56 – 155.33
5	3.33 – 28.30	3.61 – 27.59	3.38 – 27.29
6	2.91, 2.21 – 38.79	2.55, 2.37 – 36.50	2.90, 2.55 – 37.12
8	4.15 – 69.64	4.30 – 69.36	4.41 – 70.89
9	2.50 – 50.93	2.75 – 54.15	2.56 – 54.17
10	1.54 – 18.03	1.37 – 19.25	1.38 – 19.50

Table S2. 2D-HSQC assignment of the elenolic fragment for the diastereomers of monoaldehydic oleuropein aglycone using 2D-NOESY and 1D-TOCSY. C12 could not be assigned.

	**(5S,8S,9S) / (5S,8S,9R)**	**(5S,8R,9R)**	**(5S,8R,9S)**
1	9.60 – 199.96	9.81 – 201.28	9.54 – 200.19
3	7.65 – 157.37<br>7.62 – 156.08	7.65 – 157.37	7.59 – 156.08
5	3.37 – 28.30	3.62 – 27.62	3.40 – 27.11
6	2.86, 2.21 – 39.13	2.56, 2.40 – 36.61	2.88, 2.56 – 37.27
8	4.19 – 69.83	4.29 – 69.52	4.47 – 70.96
9	2.63 – 51.13	2.78 – 54.10	2.65 – 54.66
10	1.57 – 18.02	1.39 – 19.20	1.41 – 19.50

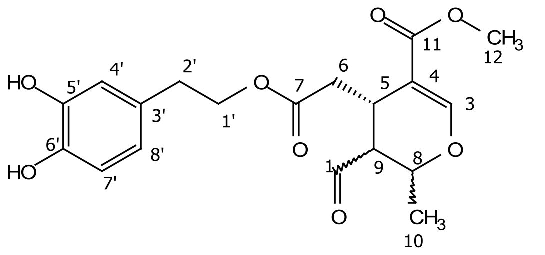

Monoaldehydic oleuropein aglycone

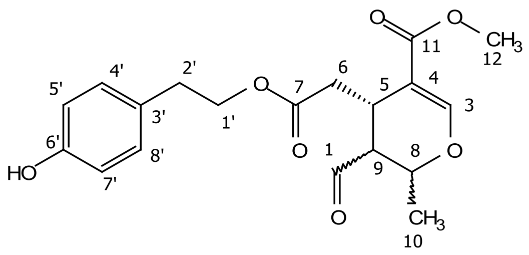

Monoaldehydic ligstroside aglycone

Figure S13. Mass spectral fragmentation of the [M−H]$^-$ ion of a) oleacein, b) oleocanthal, c) monoaldehydic oleuropein aglycone, d) monoaldehydic ligstroside aglycone and e) hydroxytyrosol-d_5. Cone voltage (V) and collision energy (eV) used for each mass spectrum are shown in parentheses.

a)

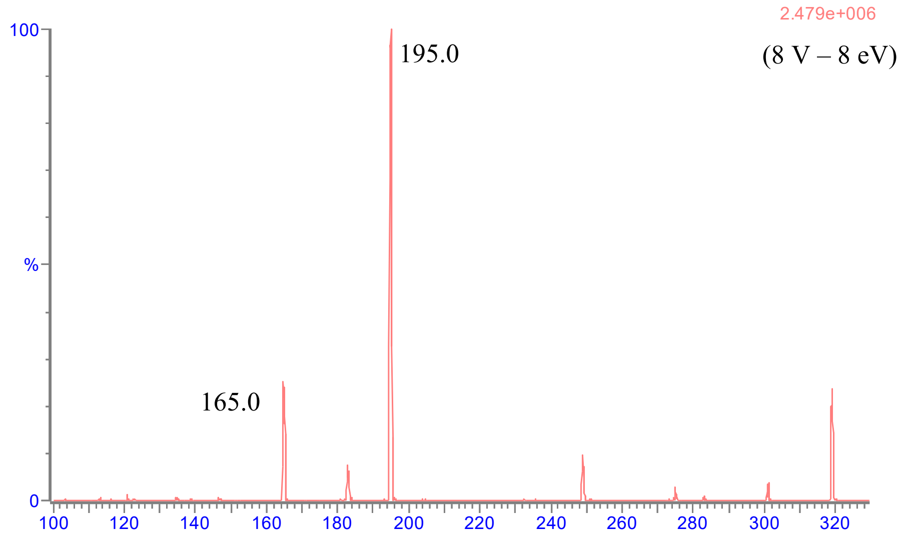

b)

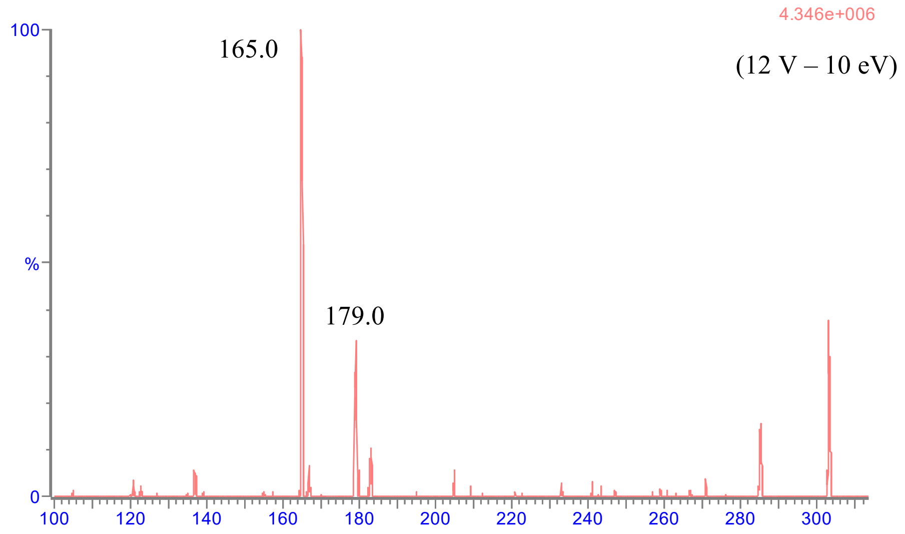

c)

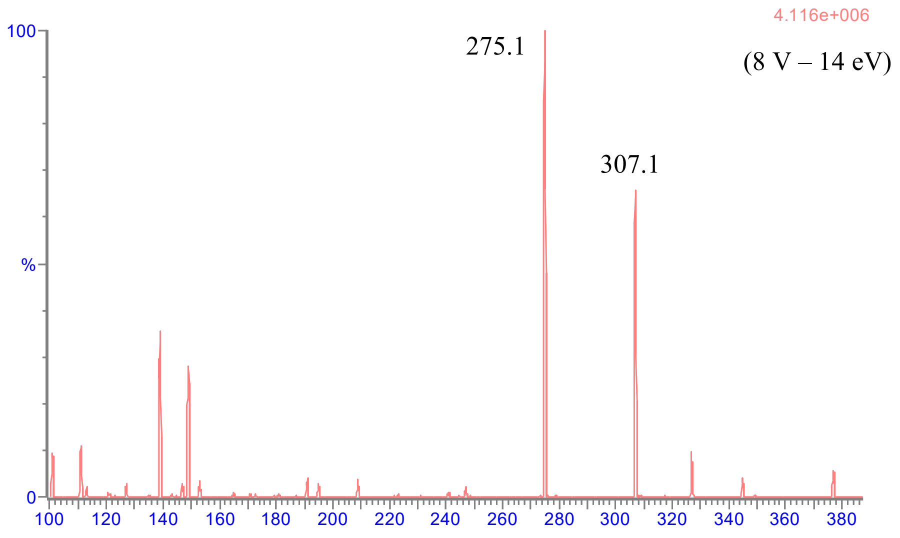
4.116e+006
(8 V – 14 eV)
275.1
307.1
100
%
0
100 120 140 160 180 200 220 240 260 280 300 320 340 360 380

d)

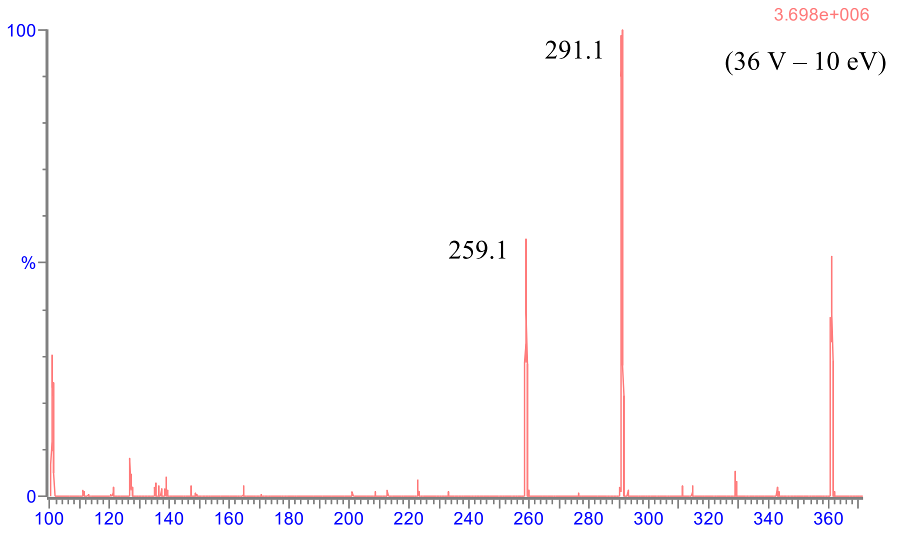
3.698e+006
(36 V – 10 eV)
291.1
259.1
100
%
0
100 120 140 160 180 200 220 240 260 280 300 320 340 360

e)

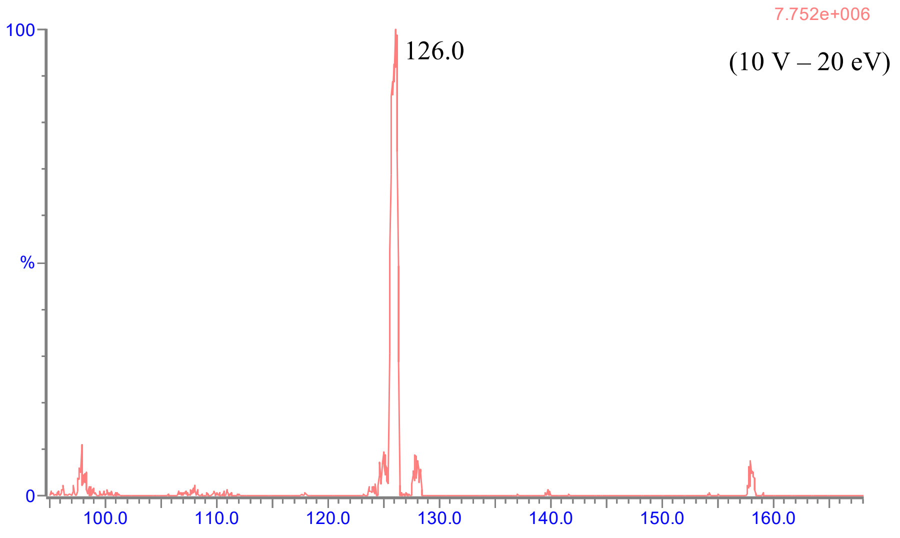
7.752e+006
(10 V – 20 eV)
126.0
100
%
0
100.0 110.0 120.0 130.0 140.0 150.0 160.0

Figure S14. Proposed fragmentation mechanisms for **3**, **4**, **5** and **6** according to the bibliography shown in Table 1.

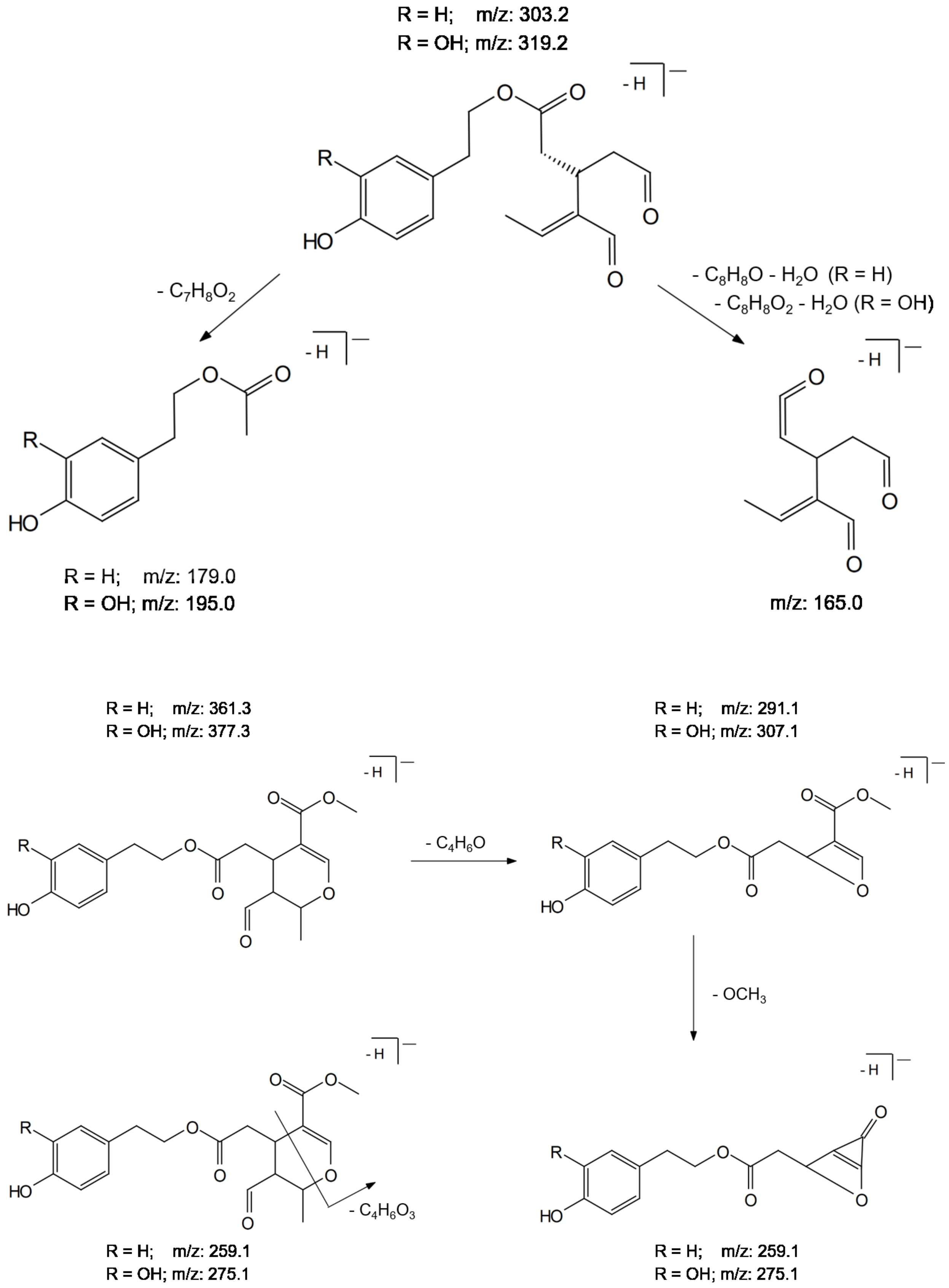

Figure S15. Chromatograms of ACN injections after analyzing a sample (V_a=25 µL; N=80). Chromatographic gradient without THF (left) and with THF (right).

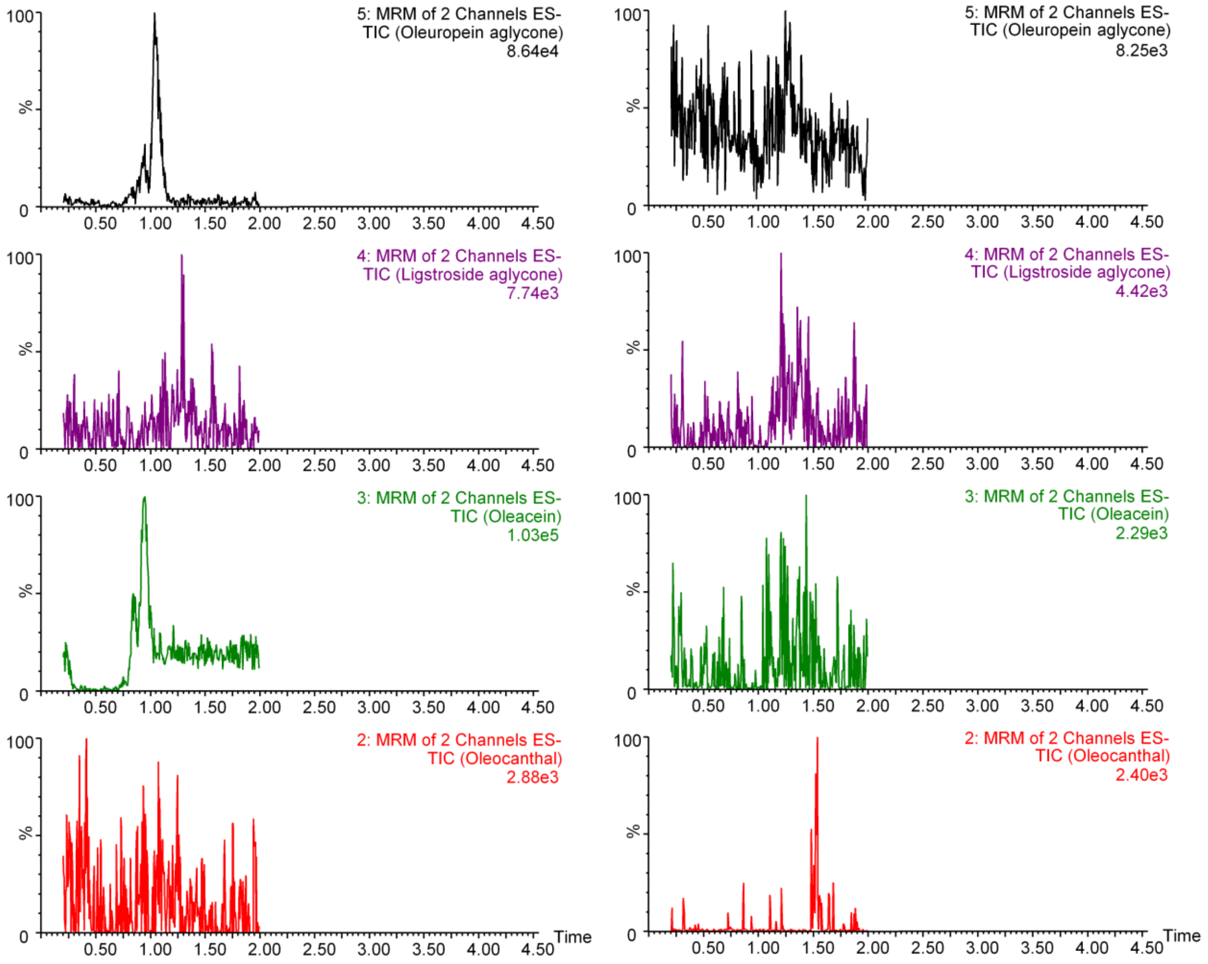

Table S3. Chromatographic program of the analytical method.

Time (min)	% A	% B	% C	
0.0	30	70	0	Isocratic separation
2.0	30	70	0	
2.1	0	100	0	Column cleaning
3.5	0	100	0	
4.0	0	0	100	
4.4	0	0	100	
4.5	30	70	0	Equilibration of column
7.5	30	70	0	

Supplementary references

1. Vougogiannopoulou, K. *et al.* One-step semisynthesis of oleacein and the determination as a 5-lipoxygenase inhibitor. *J. Nat. Prod.* **77,** 441–445 (2014).

2. Nardi, M. *et al.* Biomimetic synthesis and antioxidant evaluation of 3,4-DHPEA-EDA [2-(3,4-hydroxyphenyl) ethyl (3S,4E)-4-formyl-3-(2-oxoethyl)hex-4-enoate]. *Food Chem.* **162,** 89–93 (2014).

3. Montedoro, G. *et al.* Simple and Hydrolyzable Compounds in Virgin Olive Oil. 3. Spectroscopic Characterizations of the Secoiridoid Derivatives Gianfrancesco. *J. Agric. Food Chem.* **41,** 2228–2234 (1993).

4. Owen, R. W. *et al.* Identification of lignans as major components in the phenolic fraction of olive oil. *Clin. Chem.* **46,** 976–988 (2000).

5. Pérez-Trujillo, M., Gómez-Caravaca, A. M., Segura-Carretero, A., Fernández-Gutiérrez, A. & Parella, T. Separation and identification of phenolic compounds of extra virgin Olive Oil from Olea Europaea L. by HPLC-DAD-SPE-NMR/MS. Identification of a new diastereoisomer of the aldehydic form of oleuropein aglycone. *J. Agric. Food Chem.* **58,** 9129–9136 (2010).

6. Christophoridou, S., Dais, P., Tseng, L. I. H. & Spraul, M. Separation and identification of phenolic compounds in olive oil by coupling high-performance Liquid Chromatography with Postcolumn Solid-Phase Extraction to Nuclear Magnetic Resonance Spectroscopy (LC-SPE-NMR). *J. Agric. Food Chem.* **53,** 4667–4679 (2005).

7. Gariboldi, P., Jommi, G. & Verotta, L. Secoiridoids from Olea europaea. *Phytochemistry* **25,** 865–869 (1986).

8. Cąliş, Í., Hosny, M., Khalifa, T. & Nishibe, S. Secoiridoids from Fraxinus angustifolia. *Phytochemistry* **33,** 1453–1456 (1993).
